# reactIDR: evaluation of the statistical reproducibility of high-throughput structural analyses towards a robust RNA structure prediction

**DOI:** 10.1186/s12859-019-2645-4

**Published:** 2019-03-29

**Authors:** Risa Kawaguchi, Hisanori Kiryu, Junichi Iwakiri, Jun Sese

**Affiliations:** 1 Artificial Intelligence Research Center, National Institute of Advanced Industrial Science and Technology, Aomi, Koto-ku, Tokyo, Japan; 2 Department of Computational Biology and Medical Sciences, Graduate School of Frontier Sciences, the University of Tokyo, Kashiwanoha, Kashiwa-shi, Chiba, Japan; 3 AIST- Tokyo Tech Real World Big-Data Computation Open Innovation Laboratory, Ookayama, Meguro-ku, Tokyo, Japan; 4 Humanome Lab Inc., Shinjuku-ku, Tokyo, Japan

**Keywords:** RNA secondary structure, High-throughput structural analysis, Reproducibility

## Abstract

**Background:**

Recently, next-generation sequencing techniques have been applied for the detection of RNA secondary structures, which is referred to as high-throughput RNA structural (HTS) analyses, and many different protocols have been used to detect comprehensive RNA structures at single-nucleotide resolution. However, the existing computational analyses heavily depend on the experimental methodology to generate data, which results in difficulties associated with statistically sound comparisons or combining the results obtained using different HTS methods.

**Results:**

Here, we introduced a statistical framework, reactIDR, which can be applied to the experimental data obtained using multiple HTS methodologies. Using this approach, nucleotides are classified into three structural categories, *loop*, *stem/background*, and *unmapped*. reactIDR uses the irreproducible discovery rate (IDR) with a hidden Markov model to discriminate between the true and spurious signals obtained in the replicated HTS experiments accurately, and it is able to incorporate an expectation-maximization algorithm and supervised learning for efficient parameter optimization. The results of our analyses of the real-life HTS data showed that reactIDR had the highest accuracy in the classification of ribosomal RNA stem/loop structures when using both individual and integrated HTS datasets, and its results corresponded the best to the three-dimensional structures.

**Conclusions:**

We have developed a novel software, reactIDR, for the prediction of stem/loop regions from the HTS analysis datasets. For the rRNA structure analyses, reactIDR was shown to have robust accuracy across different datasets by using the reproducibility criterion, suggesting its potential for increasing the value of existing HTS datasets. reactIDR is publicly available at https://github.com/carushi/reactIDR.

**Electronic supplementary material:**

The online version of this article (10.1186/s12859-019-2645-4) contains supplementary material, which is available to authorized users.

## Background

RNA secondary structures play diverse roles in the fundamental RNA functions, such as gene expression regulation, translation, and localization, which are mediated by certain structural motifs or regional accessibilities [1, 2]. Due to the difficulties in the experimental determination of the secondary structures of RNAs prior to the development of the next-generation sequencing, a number of computational methods were developed to predict the secondary structures from sequences or sequence alignment data [3], but the time and computer memory requirements remain too high to be applicable at the genome-wide level [4]. To analyze the comprehensive landscape of RNA secondary structures, novel types of high-throughput experimental methods, such as PARS [5] and icSHAPE [6], have been developed using short-read next-generation sequencers, and are referred to as high-throughput RNA structural (HTS) analyses [7–9]. These methods involve the use of certain types of chemical reagents or enzymes that cause probing (e.g., modification or cleavage) at each RNA nucleotide with a different “reactivity” depending on the existence of base pairing. Using these approaches, RNA secondary structures are not directly predicted, instead, some structure-indicating scores which provide information about the molecular structures, such as reactivity scores, are obtained. The reactivity scores provide *in silico* analyses with the information necessary to guide secondary structure prediction [10], as well as to indicate the propensity of structural accessibility directly.

**Fig. 1:**
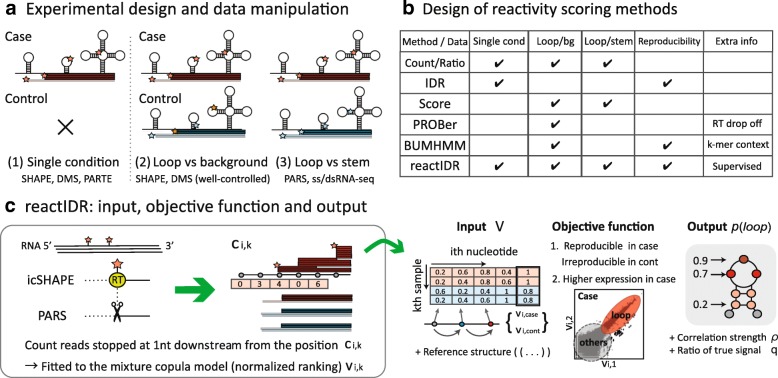
Schematic illustration of the high-throughput structural (HTS) analytical process and reactIDR. **a** Major types of HTS-associated experimental designs analyzed in this study. All experimental approaches can be classified into the listed groups, according to the number of sample conditions and their detection targets. Red and blue stars represent a probing occurrence at the loop and stem regions, respectively. **b** Applicability of the reactivity-scoring methods to three types of HTS experimental designs (i.e., whether the method is intended to analyze each type of experiment or not), in which *Score* indicates each threshold-based scoring method used for studying the icSHAPE and PARS analyses. **c** Overview of reactIDR procedures. reactIDR receives the input data consisting of 5^′^-end read-depths repeatedly measured in a single or two conditions. The input data, after trimming of the very ends of the transcripts, is converted into the scaled ranking data to adapt the copula model. Afterward, a posterior loop probability distribution at each site can be computed as an index of reactivity

As summarized in Fig. 1a, each experimental method can be used for the detection of different types of structural footprints, in order to determine the single- or double-strandedness of each nucleotide, and finally obtain the structure scores from the distribution of structural footprints. For example, the PARS method involves the activity of RNase V1 and S1, which perform structure-specific cleavage at the stem (base-paired) and loop (unpaired) regions, respectively, of the RNA molecules with a complicated structural context [5]. Conversely, using icSHAPE [6], base modifications are introduced into the loop regions by 2-methylnicotinic acid imidazolide (NAI)- *N*_3_ treatment and then detected according to a frequent drop-off at that location during reverse transcription.

While these HTS methods rely on different approaches and hold fundamental differences with regards to the structure propensity to be detected [11], they suffer from a common problem of experimental noise, such as the spurious signals that are attributed to reasons other than the experimental modifications. To reduce the misdetection of false positives, a naïve, but statistically not justified approach is the computing of the reactivity scores using the (relative) differences or ratios of the read levels between different experimental conditions, such as case/control (i.e., chemically treated samples *vs.* untreated ones) or V1/S1 treated, setting a filtering threshold for each dataset independently.

Another method for precise signal detection from sequencing read distribution is the repeated performance of experiments, since the consistency between independent replicates generally indicates the quality of the experiments beyond stochastic noise, particularly in the sequencing-based experiments [12, 13]. However, most of the existing HTS analytical approaches, including probabilistic models, such as Spats [8], ProbRNA [14], and PROBer [15], do not consider this information in order to improve the robustness of reactivity scoring. Although Mod-seeker [16] and BUMHMM [17] were developed to include the information on the reproducibility of read distribution, these and other existing probabilistic models still have issues with the high dependency on certain target experiments in their statistical or probabilistic framework, such as the empirical distribution fitted to icSHAPE-like analyses in BUMHMM. Due to the diversity of HTS methodologies, the distribution of structural footprints is highly heterogeneous (Additional file 1: Table S2 and Figures S1 and S2), and therefore, the models may not be applicable to other HTS methodologies.

The importance of comprehensive HTS data comparisons has been recently argued, in a study showing that the results obtained using different HTS approaches are less correlated than those obtained by using the same approach, and potentially contain largely non-overlapping conformational information [18]. To overcome HTS dataset heterogeneity for fair multiple experiment comparisons, one promising approach is the combining of a probabilistic model and supervised learning. Reliable structural information has been gathered in previous computational and experimental analyses [2] and many previous studies have applied supervised learning to *in silico* structure prediction to estimate the optimal model parameters [19]. However, to the best of our knowledge, there is no method that computes optimal structure scores by handling HTS raw read counts in a supervised learning manner to improve the reproducibility of HTS analyses (Fig. 1b). Therefore, computational methods for HTS analyses can be further improved, especially by developing approaches for handling multiple HTS experiments simultaneously for comprehensive understanding of the RNA secondary structure.

Here, we developed a novel method, reactIDR, to determine true reactivity signals using the replicated HTS data by calculating a statistically valid reliability score. To evaluate the reliability in a way applicable to a general HTS dataset, we extend a statistical method for chromatin immunoprecipitation (ChIP)-Seq peak detection, named irreproducible discovery rate (IDR) [13]. We applied a hidden Markov model (HMM) with the emission probability of IDR, in which the loop and stem regions are automatically segmented by a maximum posterior estimate. reactIDR is the first framework for supervised learning with known secondary structures obtained from replicated HTS sequencing data as well as applying parameter optimization based on the expectation-maximization (EM) algorithm to avoid any arbitrary step of parameter hand-tuning. According to our analyses, reactIDR has statistically one of the best accuracies for classifying loop and stem regions from HTS datasets containing multiple types of experiments. Therefore, reactIDR is suggested to be applicable to various HTS comparisons, including emerging technologies, and has the potential for increasing the value of existing HTS datasets.

## Methods

### reactIDR overview

A schematic workflow of reactIDR is presented in Fig. 1c and Additional file 1: Figure S3. We aimed to develop reactIDR so that it can be applied for solving HTS problems as follows:

- **Input** – Assuming that *K* is the number of samples in an HTS experiment, and the sequencing reads were obtained from two *K*/2 samples, under two different conditions (and an optional reference structure), each read can be mapped against a reference sequence, and the indicator of reactivity at each base can be measured by counting the reads that start at the subsequent (3^′^) base.
- **Output** – The output represents the probability that each nucleotide is the latent class *loop*.
- **Objective function** – To provide reliable structure scores for accurate structure prediction, the outputs should be optimized so that they satisfy the following conditions: (1) the sequential loop regions of the RNAs provide consistently enriched read counts under the case conditions, in contrast to those under the control conditions, while stem motifs should be enriched in the control obtained from the PARS dataset, and (2) the true signals of structural footprints should be consistent among the replicates with the higher expression.

Using the HTS approach, the positions in stem (base paired) or loop (accessible) structures can be detected by the enrichment of chemical or enzymatic footprints, since their reactivity is highly affected by the existence of base pairs. However, to remove many false positives derived from the random occurrences of fragmentation or endogenous modification, reactIDR further considers the ratio of read count enrichment between two-conditional samples. As shown in Fig. 1, *case* (*control*) samples were specifically defined as the first (last) *K*/2 samples with the sequencing reads stochastically truncated at one base downstream from the loop (stem or non-specific background, represented as bg). In reactIDR, a latent class *loop*, *stem/bg*, or *unmapped*, is expected to be assigned to the region corresponding to the case-derived, control-derived, and no sufficient reads obtained, respectively. Here, icSHAPE case and control samples were regent-treated and untreated, respectively, while PARS samples were treated with S1 and V1, respectively, and the control samples were not the untreated samples.

### IDR

Our novel algorithm, reactIDR, extends the idea of the IDR [13] criteria to obtain reproducible and irreproducible classification. We first introduce the model for the IDR estimation. Here, as an input of IDR and reactIDR, we considered the read-count data obtained for a single transcript or concatenated multiple transcripts with the total length of *L* nucleotides. As these data were initially converted into the ranking data across all positions in each experiment, these ranking data in an ascending order were scaled to the range of [0,1]. These normalized data were hypothesized to correspond to the cumulative probability distribution in the copula model, necessarily within that range. After scaling, a vector of the ranks of read counts at the *i*th position was defined as **v**_*i*_:={*v*_*i*,1_, …, *v*_*i*,*K*_} for IDR, and **v**_*i*_:={**v**_*i*,*case*_,**v**_*i*,*cont*_}={{*v*_*i*,1_, …,*v*_*i*,*K*/2_},{*v*_*i*,*K*/2+1_… *v*_*i*,*K*_}} for reactIDR. The overall input data of the IDR and reactIDR were: *V*={**v**_1_, …, **v**_*L*_}. IDR is based on the use of the mixture copula model to discriminate between one reproducible (represented by *rep*) distribution and the irreproducible (*irep*) distributions, recognizing them as true and false signals, respectively. Each copula can explain the distribution of true, reproducible or false, poorly correlated, signals. This is because true signals obtained from the protein binding sites in ChIP-Seq should exhibit reproducible enrichment with an increase in the read-count depth and a high correlation between the replicates. Based on this, the signals categorized into *rep* are thought to contain many more true signals compared with those in the *irep* group.

For simplicity, we considered an example of the mixture of two Gaussian copulas in two dimensions hereafter, showing that our approach can be used for the analysis of two experimental replicates. When the *i*th signal is produced by true (false) signals, represented as *r*_*i*_=*true*(*false*), the distribution of a random variable *x*_*i*_=(*x*_*i*,1_,*x*_*i*,2_) corresponding to the *i*th signal is as follows:

1 $$ \begin{aligned} \left(\begin{array}{c} x_{i,1} \\ x_{i,2} \end{array}\right) {\Bigg\rvert}{h} \sim \mathcal{N} \left(\left(\begin{array}{c} \mu_{h} \\ \mu_{h} \end{array}\right),\left(\begin{array}{cc} \sigma_{h}^{2} & \rho_{h} \sigma_{h}^{2} \\ \rho_{h} \sigma_{h}^{2} & \sigma_{h}^{2}, \end{array}\right)\right)  \end{aligned}  $$

where *μ*_*h*_, *σ*_*h*_, and *ρ*_*h*_ represent mean, variance, and correlation, respectively, with the indicator *h* for each state of *r*_*i*_ (*h*=1 when *r*_*i*_=*true* and *h*=0 when *r*_*i*_=*false*). The distribution of true *rep* signals is assumed to contain a positive mean and correlation (*μ*_1_>0 and 0<*ρ*_1_≤1), while spurious *irep* signals should be located around the low-coverage regions and show increased variance ($\mu _{0}=0, \ \sigma _{0}^{2} = 1$, and *ρ*_0_=0). These parameters can be fitted not to the actual read counts, but to the normalized ranks of read coverage for ChIP-Seq data **v**, such that those ranks are derived from the cumulative marginal probability distribution function of *x*_*i*,*k*_ (*k*=1,2), as shown below:

$${\begin{aligned} F_{k}(x_{k}) := \ v_{i, k} = {\int}_{-\infty}^{x_k} q\mathcal{N}\left(x^{\prime}_{k}|\mu_{1}, \sigma_{1}^{2}\right)+(1-q)\mathcal{N}\left(x^{\prime}_{k}|\mu_{0},\sigma_{0}^{2}\right) dx^{\prime}_{k}, \end{aligned}} $$

where *q* represents the ratio of true and reproducible signals in the samples and *k* represents the *k*-th experiment. By considering the contribution of the second Gaussian distribution representing irreproducible signals, a probability of each peak derived from the irreproducible signal or IDR, can be estimated.

### reactIDR: an algorithm based on the IDR and HMM

**Fig. 2:**
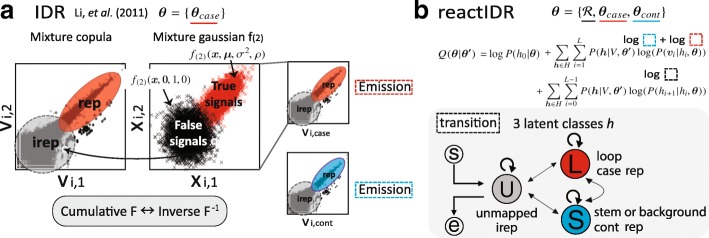
Comparison between the irreproducible discovery rate (IDR) and reactIDR model. **a** IDR is a method used for the classification of signals into true (high-coverage and reproducible) and false (low-coverage and irreproducible) signals, based on the Gaussian mixture model, by associating the observation with the cumulative joint probability distribution and pseudo-value *x*. **b** In reactIDR, IDR is combined with the hidden Markov model of the three latent classes: *loop*, *stem/bg*, and *unmapped*. The start and end of transcripts should be assigned to the *unmapped* class. These three classes are defined to handle case/control comparisons with the Gaussian mixture copulas for each condition simultaneously, in which each parameter is optimized to the data and reference structure using the expectation-maximization algorithm

While IDR is a powerful method for the evaluation of the reliability of various joint distributions of ChIP-Seq peaks, HTS data used for RNA stem/loop detection must additionally include the consideration of the dependency between the consecutive nucleotides over multiple conditions. The application of IDR to HTS analyses, therefore, requires further specific extensions for the more complicated situations. To this end, we developed reactIDR, a novel method for the extraction of true reactivity signals from the replicated HTS data, based on the determination of statistical reliability score according to the IDR and HMM (Fig. 2).

For *L* as a total length of transcripts and a vector of scaled rankings at the *i*th position, *v*_*i*_ can be given. In reactIDR, a latent variable at the *i*th position *h*_*i*_ can belong to any element of {*loop*, *stem/bg*, and *unmapped*}, corresponding to each status associated with the HTS data and RNA secondary structures. The enrichment of *v*_*i*_ is thought to be most likely observed at the regions that belong to the *loop* class in the case samples and *stem/bg* class in the control samples, while we did not expect any specific enrichment in the case of *unmapped* classes.

In reactIDR, the paths of all latent variables can be represented by **h**={*h*_0_, …, *h*_*L*_}, and the likelihoods of *V* and **h** can be obtained as the products of emission and transition probabilities, as formulated below:

2 $$ \begin{aligned} P(V, \mathbf{h}|\mathbf{\theta}) =& P\left(h_{0}|\mathbf{\theta}\right) \prod\limits_{i=1}^{L} P\left(\mathbf{v_i}|h_{i},\mathbf{\theta}\right) \prod\limits_{i=0}^{L-1}P\left(h_{i+1}|h_{i}, \mathbf{\theta}\right)  \end{aligned}  $$

where **θ** consists of the transition matrix between each latent class pair $\mathcal {R}$, and the set of all of parameters in the copula model for case samples *θ*_*case*_ and control samples *θ*_*cont*_, respectively (i.e., **μ**, *σ*^2^, and *ρ*, see Additional file 1: Figures S4 and S5). Due to the difficulties in the mapping of reads into the edge of transcripts, *h*_0_ and *h*_*L*_ are always assigned to the *unmapped* class (*P*(*h*_0_|**θ**)=1 and *P*(*h*_*L*_|**θ**)=1). *P*(*h*_*i*+1_|*h*_*i*_,**θ**) represents the transition probability between *h*_*i*_ and *h*_*i*+1_ in the HMM, represented by $\mathcal {R}$, which is further optimized by the expectation of the transition between *h*_*i*_ and *h*_*i*+1_ at each step of the EM algorithm iteration. *P*(**v**_*i*_|*h*_*i*_,**θ**) is an emission probability, which we defined as follows:

$$\begin{aligned} P\left(\mathbf{v}_{i}|h_{i},\mathbf{\theta}\right) =& P\left(\mathbf{v}_{i, case}|r_{{case}}, \mathbf{\theta}_{{case}}\right) \cdot P\left(\mathbf{v}_{i,cont}|r_{{cont}}, \mathbf{\theta}_{{cont}}\right)\\ r_{{cont}} :=& \ rep \text{ if}\ h_{i} =stem/bg \text{ and}~ irep \text{ otherwise} \\ r_{{case}} :=& \ rep \text{ if}\ h_{i} =loop \text{ and}~ irep \text{ otherwise }, \end{aligned} $$

where **v**_*case*_ and **v**_*cont*_ are expected to show the enrichment of loop-like and stem-like (or background noise) regions, respectively. These probabilities for *rep* and *irep* signals can be obtained by the mixture Gaussian copula, as when using the IDR.

Joint distribution *f*_(2)_, cumulative marginal distribution *F*_*k*_(=*v*_*i*,*j*,*k*_), and the function of the vectorized inverse of *F*_*k*_*F*^−1^ can be defined as follows:

$$\begin{aligned} f_{(2)}\left(\mathbf{x}, \mathbf{\mu}, \sigma^{2}, \rho\right) =& \mathcal{N}\left(\mathbf{x}| \mathbf{\mu}, \sigma^{2}, \rho\right) \\ F_{k}\left(x_{j,k}\right) =& {\int}_{-\infty}^{x_{j,k}} f_{k}\left(x^{\prime}_{k}\right)dx^{\prime}_{k} \,=\, {\int}_{-\infty}^{x_{j,k}} q_{j}\mathcal{N}\left(x^{\prime}_{k}|\mu_{h}, \sigma_{h}^{2}\right)\\ &+\left(1-q_{j}\right)\mathcal{N}\left(x^{\prime}_{k}|\mu_{0},\sigma_{0}^{2}\right) dx^{\prime}_{k} \\ F^{-1}\left(\mathbf{v_{j}}\right) =& \left\{ F^{-1}_{1}\left(v_{i, j, 1}\right), F^{-1}_{2}\left(v_{i, j, 2}\right) \right\}, \end{aligned} $$

where *h*=1 (2) when *j*=*case* (*cont*), and *q*_*j*_ represents the ratio of true signals in *j* samples. Afterward, the emission probability based on the mixture Gaussian copula consisting of *rep* and *irep* distributions with parameters **θ**_*case*_ or **θ**_*cont*_ can be formulated as follows:

$$\begin{aligned} P\left(\mathbf{v}_{i, j}| \ r_{j}, {\boldsymbol{\theta}_{\boldsymbol{j}}}\right) =& \frac{f_{(2)}\left(F^{-1}\left(\mathbf{v}_{i,j}\right), {\boldsymbol{\mu}_{\boldsymbol{h}}}, \sigma_{h}^{2}, \rho_{h}\right)}{ \prod_{k = 1}^{2} f_{k}\left(F^{-1}_{k}\left(v_{i,j,k}\right)\right)} & \text{ if } r_{j} = \text{ rep} \\ =& \frac{f_{(2)}\left(F^{-1}\left(\mathbf{v}_{i,j}\right), \mathbf{\boldsymbol{\mu}_{\boldsymbol{0}}}, \sigma_{0}^{2}, \rho_{0}\right) }{ \prod_{k= 1}^{2} f_{k}\left(F^{-1}_{k}\left(v_{i,j,k}\right)\right)} & \text{ otherwise,} \end{aligned} $$

where *μ*_*k*_>0 and 0<*ρ*_*k*_≤1 while $\mu _{0}=0, \ \sigma _{0}^{2} = 1$, and *ρ*_0_=0, the same as those in Eq.1 (further details can be found in Additional file 1). In this way, using reactIDR, we can compute a posterior probability distribution for each latent class at each site as an index of the reactivity and evaluate its reliability. Using the EM algorithm, each parameter can be iteratively optimized to maximize the expectation of likelihood Eq. 2 in reactIDR. Furthermore, reactIDR can incorporate supervised learning at this step, by limiting *H* so that it is consistent with the reference structure. Specifically, *loop*, *stem/bg*, and *unmapped* class is associated with loop, stem, and regions, respectively, in which *stem/bg* class is expected to play a role in removing false positives from true loop regions in the icSHAPE datasets. Afterward, trained parameters were used as the initial set of parameters in the re-fitting process for each rRNA sequence of the test set. The details of the optimization process, such as the derivative of *Q* for each parameter, are described in Additional file 1.

### Datasets used and the evaluation of classification based on reactivity indicators

In this study, datasets generated by using two HTS methods, icSHAPE and PARS, were used to validate the accuracy of reactIDR. A whole-transcriptome PARS dataset of the native deproteinized transcriptome of GM12878 cells in vitro was obtained (GEO accession number, GSE50676) [20]. Here, we used the normalized read counts of two replicates treated with nucleases S1 and V1 (hereafter referred to as S1 and V1, respectively). Analyses were also conducted using the icSHAPE dataset obtained for the HEK293T cells (GEO accession number, GSE74353), which contains sequencing reads obtained for three conditions with two replicates: dimethyl sulfoxide (DMSO)-, in vitro NAI-N3-, and in vivo NAI-N3-treated cells [21]. Computation of the original PARS and icSHAPE scores and the characteristics of these datasets can be found in Additional file 1: Section 2 and Figures S6 and S7.

To construct the reference set of the rRNA sequences, a human ribosomal repeating unit (NT_167214.1) was extracted from the NCBI database, and a 5S rRNA sequence (ENST00000364451) was additionally included. As a rRNA structure reference, cryo-EM-based ribosomal structure (PDB ID: 4v6x) was aligned to our reference sequence [22]. To obtain base-to-base correspondence between the reference sequences and the structure, 5S, 5.8S, 18S, and 28S rRNA reference sequences were aligned to each sequence within the structure dataset, and all bases successfully aligned to the reference were used for the evaluation of classification. Of the four rRNAs in the cryo-EM ribosomal structure, 18S rRNA reference structure was used as the training set for the supervised learning of reactIDR, with the negative alignment data for the minus strand of each rRNA, representing the read counts produced by misalignment. The accessible surface area (ASA) of the ribosome structure was calculated for each nucleotide using NACCESS with default parameter settings [23].

To evaluate the accuracy of structure classification based on reactivity indicators, we constructed a receiver operating characteristic (ROC) curve. In the ROC curve, the y-axis corresponds to the true positive rates, while the x-axis corresponds to the false positive rate. *P*-values were computed in order to compare the area under curve (AUROC) of reactIDR and other scoring methods by using the pROC library in R with a bootstrap method with 100 repetitions. We measured the accuracy of *in silico* structure prediction with or without the structure scores by positive predictive value (PPV) and sensitivity (identical to the true positive rates) for each possible base pair within the transcript except for pseudo-knots. The accuracy of the structure predictions based on multiple HTS datasets was investigated using the support vector machine (SVM) as well, through the scikit-learn library interface. For *in silico* structure prediction assisted by the structure scores, RNAfold from the Vienna RNA package v2.4.0 [3] was applied.

## Results

### Characteristics of the IDR-based structure classification with or without HMM

**Fig. 3:**
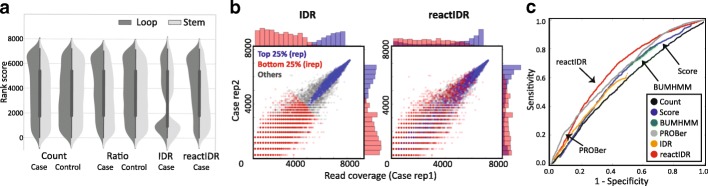
Distribution of raw read counts and structure scores obtained using reactIDR at stem and loop loci. **a** Violin plots, showing the ascending rank orders of the four indices of chemical footprint enrichment that can be computed for four rRNAs from case and control samples separately: *Count*, raw read count; *Ratio*, read count normalized by the reads passing through the position; irreproducible discovery rate (IDR); and reactIDR. With the increase in the rank of the structure score obtained from the case samples, the likelihood of the nucleotide being in the loop structure increases. **b** Read count distribution of reproducible and irreproducible groups, classified into top 25*%* and bottom 25*%* rankings, based on the IDR (left) and reactIDR reactivity indicator(right) criteria. **c** Receiver operating characteristic (ROC) curve, showing stem/loop structure prediction of the test set which is composed of 5S, 5.8S, and 28S rRNAs based on the structure scores obtained using six scoring methods, for the in vivo icSHAPE dataset

We investigated the improvements that can be obtained by combining HMM with IDR as criteria for stem/loop classification in reactIDR. To compare the read coverage distribution of HTS analysis between the case/control conditions and stem/loop positions, in vivo icSHAPE data were aligned to the reference sequence of rRNAs and read count (*Count*) was obtained for duplicated case and control samples. The structure scores based on the *Ratio* (normalized read count ratio versus the number of reads passing through that position), IDR, and reactIDR were also computed from the distribution of *Count* scores. In Fig. 3a, the distributions of the rank orders from the indices of chemical footprint enrichment for case and control samples, and stem and loop locus, individually, are presented. The locations of loop and stem structures were predicted for four human rRNAs using RNAview [24] for the 3D structure of ribosome determined by cryo-EM (details can be found in Additional file 1). The distributions of averaged *Count* and *Ratio* rank scores of the treated samples were shown to contain a larger number of high-ranking scores to a similar extent each other, compared with those of the control samples. The distribution of the IDR rank scores demonstrated two separate clusters associated with the reproducibility due to the characteristics of two Gaussian mixture copula models (Fig. 3b). Using reactIDR, more distinguishable distributions of the higher-ranked scores at the loop regions were obtained, compared with those at the stem regions. This may be because the structure scores obtained with reactIDR were estimated based on the case/control ratios and 18S rRNA reference structure, while other three indices were used only for the evaluation of the read count enrichment of the samples belonging to one condition.

Since the top rankings of reactIDR tended to contain the nucleotides with not only high read coverage (Fig. 3b), using reactIDR, we were able to fit more complicated enrichment patterns considering the read coverage of surrounding nucleotides. Moreover, even for the noisy genome-wide analysis of the PARS data, IDR-based classification was successful as well in associating the reproducibility of read enrichment and the strength of stem probability obtained *in silico* (Additional file 1: Figure S8). Taken together, using reactIDR case and control read enrichment criteria and employing the data showing the reproducibility and reference structure, we were able to infer the accessibility of each nucleotide with higher precision than that possible when using the raw read count.

### Comparison of reactIDR structure classification accuracy for 2D accessibility

To evaluate the accuracy and robustness of reactIDR for different HTS methodologies, stem/loop classification based on the structure scores using multiple HTS rRNAs datasets was performed. For a fair comparison, only 18S rRNA data were subjected to parameter optimization using reactIDR and other rRNAs were subjected to comparison. In addition to the structure scores used in the previous analyses, three more types of reactivity scoring methods were computed for each dataset: *Score* used in the original HTS studies, BUMHMM [17], and PROBer [15]. Afterward, the AUROCs were computed for rRNA structure classification, while the structure status expected to be enriched (i.e., stem or loop) was set to positive. AUROCs for each classification obtained using icSHAPE and PARS datasets are shown in Table 1. reactIDR was observed to generate the highest AUROC for the icSHAPE datasets and only the prediction accuracy of PROBer was statistically comparable to the reactIDR results obtained using in vivo and in vitro icSHAPE datasets. This consistency was also confirmed for different choices of the training set as well (Additional file 1: Tables S3 and S4). In Fig. 3c, ROC curves of six structure score-based predictions for the in vivo icSHAPE dataset are presented (see also Additional file 1: Figures S9 and S10). Using reactIDR, we achieved the highest accuracy for the medium portions (0.1−0.6) among the overall range of 1-specificity values, shown along the x-axis. For the stem (V1) prediction from the PARS datasets, reactIDR generated the highest AUROC among all reactivity scoring methods as well. In contrast, the prediction accuracy of all available methods was shown to be lower than that randomly obtained for the PARS S1 dataset, unless the common structure scores were computed for the stem/loop predictions using the S1 and V1 (*Score*, BUMHMM, and PROBer). As the enrichment of the read count was not observed at loop regions in the PARS S1 dataset (Additional file 1: Figure S11), the PARS S1 dataset of rRNAs itself is suggested not to follow the experimental hypothesis.

**Table 1:** Area under the ROC curve (AUROC), showing stem/loop classification of reactivity scoring methods

	icSHAPE		PARS	
Method	in vivo	in vitro	V1(cont)	S1(case)
Count	*0.555	*0.514	* 0.702	* 0.349
Score	*0.600	*0.564	*0.574	
BUMHMM	*0.592	*0.526	*0.634 *†*	
PROBer	0.645	0.607	*0.639 *†*	
IDR	*0.563	*0.518	* 0.664	* 0.375
reactIDR	**0.665**	**0.626**	**0.715**	0.420

* AUROC is significantly lower than that of reactIDR with *p*-value<0.05 for the comparison after Bonferroni correction. *†* AUROCs obtained by methods not originally designed for PARS experiments. Structure scores of *Score*, *BUMHMM*, and *PROBer* are common for the case and control conditions of the PARS dataset. Their AUROC significances were computed for that of reactIDR for the PARS V1 condition

**Fig. 4:**
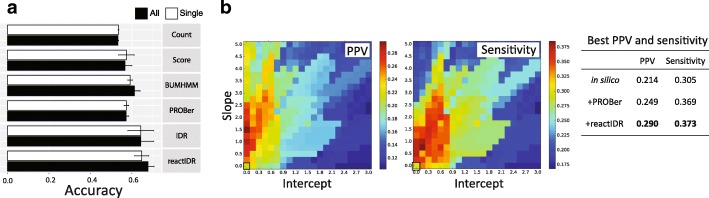
Application of structure scores for structure prediction using a machine learning approach and *in silico* pseudo-energy model [10] for the test set of three rRNAs. **a** Stem/loop structure prediction accuracy based on the structure scores computed from individual or combined datasets (icSHAPE in vivo and in vitro and PARS datasets) by the support vector machine, with the removal of indeterminable scores. Average and variance of accuracy were computed after 10-fold cross-validation. The predictions based on a single dataset utilized the structure scores from either in vivo icSHAPE dataset, in vitro icSHAPE dataset, or PARS V1 dataset, and the results of the maximum mean accuracy are presented. **b** Positive predictive value (PPV) and sensitivity of *in silico* structure prediction using RNAfold with and without reactivity assisting. The best PPV and sensitivity pair was selected to maximize their sum for each prediction condition

Moreover, we examined the accuracy of the structure scores from multiple HTS datasets combined with SVM and linear discriminant analysis, a representative machine learning method. Using the same training and test set of rRNAs, reactIDR showed the highest mean prediction accuracy compared with that obtained for the other methods by merging the features of structure scores for all datasets (Fig. 4a and Additional file 1: Figures S12 and S13). These results suggest the robustness of reactIDR when used in combination with different HTS approaches, and the potential of its use for the integrative analyses of various HTS datasets, regardless of the classification method. The reactIDR reliability was further examined for *in silico* structure prediction, particularly for base pair prediction, using RNAfold [3]. We applied one of the previously developed pseudo-free energy methods [10] to perform a hybrid structure prediction of computational and experimental structural analyses, in which the reactivity is accounted for in the log linear form as *ΔG*_SHAPE_=*m* ln(reactivity+1)+*b*. In Fig. 4b, PPV and sensitivity of the minimum free energy structure predicted by RNAfold with structure scores of reactIDR are shown, with the progressive changes in the parameters of the slope *m* and intercept *b*. The structure scores of reactIDR with RNAfold were shown to perform better than *in silico* prediction and that for PROBer based on the best PPV and sensitivity pair selected to maximize the sum of pair (Fig. 4b and Additional file 1: Figures S14 and S15).

Taken together, the reactIDR scores were able to improve the prediction accuracy of integrative analyses as well as *in silico* structure prediction as conformational constraints. According to the assumptions used for reactIDR, the output structure scores of reactIDR correspond to the probability of the signals being true or false signals, which accounts for the reproducibility. Therefore, it may be theoretically applicable to the cross-comparison of exact reactivity scores between the datasets obtained by different HTS methods to combine them for further structure prediction, such as pseudo-free energy methods [10] and recently developed machine learning methods [19].

### Correlation between RNA reactivity and 3D accessibility

**Fig. 5:**
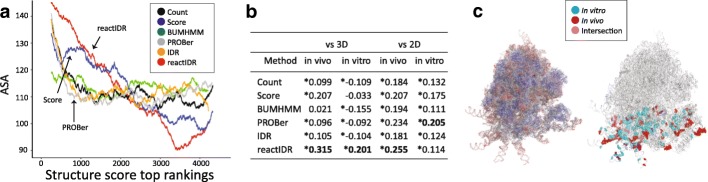
Correlation between RNA reactivity and 3D accessibility for the test set of three rRNAs. **a** Relation between the accessible surface area (ASA) and reactivity scores computed by six types of scoring methods. y-axis, averaged ASAs within each 500-nucleotide sliding window; x-axis, a ranking of each nucleotide based on the structure scores, in which ties were randomly broken. The structure scores of reactIDR from the in vivo icSHAPE dataset presented the highest correlation with ASAs across the six scoring methods. **b** Correlations between structure scores and 2D or 3D accessibility indices. * *p*-value<10^−5^ after Bonferroni correction. **c** Ribosomal 3D structures visualized by PyMOL [29]. Left, ribosome changes its color from red to blue according to the ASAs of each nucleotide (high to low). Right, the highlighted regions indicate the top 7*%* reactive sites of 18S rRNA from the in vivo dataset (red), in vitro dataset (blue), and their intersection (purple)

The agreement between reactivity and 2D accessibility was demonstrated for rRNAs, but the reactivity of RNA in a chemical probing reaction appears to be affected by the 3D conformation, as observed in some previous studies [25], in particular, the relationship between the presence of the sugar puckers of RNA structures in *C*2^′^- or *C*3^′^-end and the modification efficiency of SHAPE reagent [26, 27]. To assess the influence of 3D accessibility on reactivity, we examined the correlation of the structure score with 2D and 3D accessibility, using ASA. In Fig. 5a, the relationship between ASA and structure score is presented, in which ASA is averaged with the 500-nt sliding window estimates for the test set of rRNAs from the in vivo icSHAPE dataset (Additional file 1: Figure S16). All analyzed methods showed a positive correlation between ASAs and structure score ranking (Fig. 5b), with the exception of a partial region of ties that appeared when using the BUMHMM or PROBer. Furthermore, using reactIDR, the highest correlation was obtained across six scoring methods with a statistically significant *p*-value (*p*<10^−10^). This result is consistent with the high accuracy of stem/loop classification because 2D accessibility and 3D accessibility are highly correlated as well (Additional file 1: Figure S17). On the other hand, the correlation between structure scores and 3D accessibility was lower for the in vitro dataset, except for the moderate decline obtained when using reactIDR, with the subtle differences in the correlation between structure scores and 2D accessibility obtained when setting the loop and stem structures to 1 and 0.

The in vivo reactive sites (represented in red in Fig. 5c) were observed to be located at the outer side of ribosomes, unlike those obtained in vitro, and the regions with the highly reactive nucleotides in vivo and in vitro rarely overlapped, suggesting that the 3D conformation affects the results obtained when using the in vivo dataset. Since the aim of reactIDR is to allow comparisons between different HTS datasets in a fair manner, the reactIDR structure scores can potentially be used for the quantification of conformational changes between different conditions.

## Discussion

In this study, reactIDR was shown to achieve an accuracy statistically comparable to that of PROBer (Fig. 3c and Additional file 1: Figure S10). Although reactIDR uses several assumptions for the analysis of HTS datasets, such as rank-based read count evaluation, localization of stem and loop loci, and the arbitrary assignment of latent classes during the supervised learning, our results suggest the robustness and rationality of reactIDR application to the HTS datasets (Additional file 1: Section 1.9).

To examine the robustness of reactIDR, we further examined the structure prediction of reactIDR from the SHAPE and mutational profiling (MaP) datasets obtained in [30], in which the reactivity was measured by capillary electrophoresis. In spite of the severe differences in experimental techniques, reactIDR still showed high and stable AUROCs (>0.8) for *E. coli* rRNAs with the lower variances compared to the case using the average of the original reactivity scores (Additional file 1: Figure S18).

Importantly, although the increase of AUROCs was not substantial for its computational time (Additional file 1: Table S1), reactIDR is still considered to possess the advantage of helping researchers to set a reasonable threshold based on the probability being irreproducible. For example, when the threshold of reactIDR is set to 0.05 for the SHAPE-MaP dataset, true and false positive rate of the loop prediction is 0.81 and 0.39, respectively. Moreover, reactIDR is expected to be applicable to the heterogeneous HTS datasets which are known to have a specific bias by using post-processed scores after removing the biases. As such, further extensions can be considered to increase the applicability and flexibility of reactIDR for a variety of data, such as accounting for the base type dependency that clearly appeared in DMS-Seq [28] as well as further optimization for the HTS datasets without any replicates or more complicated structural footprints base-pair detection by RNA crosslink [21].

## Conclusions

We have developed a novel software, reactIDR, for the prediction of stem/loop regions from the HTS analysis datasets, based on the reproducibility criterion. For the rRNA structure analyses, reactIDR was shown to have robust accuracy across different datasets. Moreover, the structure scores obtained with reactIDR enhanced the prediction accuracy by integrating multiple datasets and *in silico* structure analyses. Considering the 3D accessibility, reactIDR structure scores showed the significant and highest correlation, suggesting the potential of structure scores to reflect both 3D and 2D accessibility. Since reactIDR is the first method for the comparison of the HTS datasets obtained from multiple sources in a single unified model, it may help increase the accuracy of the RNA secondary structure predictions at transcriptome-wide level, allowing further studies.

## Additional file

Additional file 1 The detail of mathematical background, experimental design, and results of additional experiments. (PDF 1680 kb)

## Abbreviations

IDR: irreproducible discovery rate
HMM: hidden Markov model
HTS: high-throughput structural
EM: expectation-maximization
ROC: receiver operating characteristic
AUROC: area under the receiver operating characteristic curve
SVM: support vector machine
PARS: parallel analysis of RNA structure
SHAPE: selective 2^*′*^-hydroxyl acylation analyzed by primer extension
DMS: dimethyl sulfate
MaP: mutational profiling
NAI: 2-methylnicotinic acid imidazolide
