# Supplementary material for: reactIDR: evaluation of the statistical reproducibility of high-throughput structural analyses towards a robust RNA structure prediction
Source: BMC Bioinformatics. 2019 Mar 29;20(Suppl 3):130. doi: 10.1186/s12859-019-2645-4 (PMC6439966; doi:10.1186/s12859-019-2645-4)
Supplement: Supplementary file 1 — The detail of mathematical background, experimental design, and results of additional experiments. (PDF 1680 kb) [file 12859_2019_2645_MOESM1_ESM.pdf]

reactIDR: evaluation of the statistical reproducibility of high-throughput structural analyses towards a robust RNA structure prediction

Risa Kawaguchi, Hisanori Kiryu, Junichi Iwakiri, and Jun Sese

February 1, 2019

1 reactIDR: A novel algorithm for extracting reproducible signals based on IDR and hidden Markov model

A novel pipeline, reactIDR is designed to evaluate the reliability of structure scores from high-throughput structural analysis (HTS analysis) with the criteria of reproducibility. To compute reliability of reactivity indicators, we developed a novel algorithm for the case/control comparison of HTS datasets. reactIDR is based on the combined model of Hidden Markov Model (HMM) and Irreproducible Discovery Rate (IDR) [1] and iteratively optimizes model parameters by EM algorithm. reactIDR can also perform a supervised learning, optimizing the model parameters if known reference structures are given. In the following subsection, we describe the algorithm of reactIDR as well as the basic idea of IDR.

1.1 Input data of high-throughput structural analysis

To determine RNA secondary structure in transcriptome level, tens of HTS methods have been actively developed by combining RNA structure-specific probing and short read sequencing techniques. In probing-based HTS methods such as SHAPE-Seq [2] and DMS-Seq [3], the base modification induced at flexible and reactive nucleotides increases the drop-off ratio of reverse transcription (RT) on the spot. This phenomenon induces an enrichment of 5′-ends at a single base downstream of accessible bases after RT. Cleavage-based HTS methods such as PARS [4] and ds/ssRNA-Seq [5] also measures the same enrichment as the strength of single- or double-strandedness, depending on a treated nuclease. For example, nucleases S1 and V1, applied in PARS, have been used to cause cleavage at loop and stem structures, respectively. Note that nuclease S1 and V1 have individual characteristics and limitation to recognize base pairs, as a cleavage of V1 is suggested to show a preferential recognition for the tips of helices rather than the inside of helices [6]. Among such many HTS methods applicable for transcriptome, there is still a common problem of systematic biases, such as differences of RNA expression or gradual decrease of sequencing read from 3′- to 5′-end. To solve such problem, some previous studies take additional information into accounts such as total read counts aligned to each transcript or elongated cross over each modification [5]. However, there is no perfect method to reduce the influence of noises sufficiently so far.

In this study, a typical input data from HTS is assumed to be the set of coverage profiles of sequencing reads whose 5′-ends are located at a single base downstream of each position as

shown in Figure 1 of the main paper. Those profiles are represented by "read count", "read depth", or "read coverage" information. Moreover, reactIDR can handle various types of read count information such as read count normalized by the reads passing through the position, or reactivity scores computed by any existing method. In this study, however, reactIDR was simply applied to the datasets of raw read counts.

Let there be read coverage data of K samples from HTS. IDR computation is only applicable to K samples in a single condition and it requires certain preprocessing for case/control comparison. On the other hand, reactIDR is applicable to the case of the replicate samples associated with two different conditions, such as reagent treated/untreated samples in SHAPE-Seq-like methods and nuclease S1/V1 treated samples in PARS experiments. For simplicity, we define these two conditions as *case* and *control*, respectively. It should be emphasized that the *case* and *control* samples in this study do not necessarily correspond to truly case and control samples from the aspects of each experimental methodology. This is because reactIDR aims to extract the significant enrichment of read counts obtained for only one of the conditions compared to others.

Here, we introduce some notations for the description in the following subsections. For the ith position of transcript t, the total of mapped reads $c_{t,i,k}$ is obtained from the dataset at the kth experiments among the K experiments. Because IDR estimation is performed on a Gaussian mixture copula model for the joint cumulative distribution, $c_{t,i,k}$ is first converted into a rank order $v_{t,i,k}$ scaled into the range (0, 1) in kth sample. The tied values are randomly ordered to fit a copula model, in which a marginal joint distribution is assumed to be a continuous uniform distribution without any ties. This randomness is expected to enhance the irreproducibility of low read-depth regions. Then, an input of reactIDR can be considered as a K-dimensional vector $\boldsymbol{v}_{t,i}$ for each position i and transcript t as defined below.

$$\boldsymbol{v}_{t,i} := \left\{v_{t,i,1}, \ldots, v_{t,i,K}\right\}$$

This conversion can be performed for either of each transcripts or all transcribed nucleotides. For the latter case, read counts for each transcript are virtually concatenated into an L-long sequence of a single transcript. At the same time, an appropriate transition boundary is set between the individual transcripts due to the contextual dependency assumed in reactIDR. In IDR method, there is no assumption about a contextual dependency between neighborhood bases. On the other hand, in reactIDR method, the dependency between immediate neighbors is considered in one-dimensional HMM to take into accounts the local similarity of secondary structure as well as read coverage. In this study, we applied the first conversion for the accurate prediction of well-sequenced rRNA datasets while others will be still effective for the datasets including poorly-sequenced transcripts. For that reason, a notation of the transcript t is omitted from v_i in our description, hereafter. The whole set of v_i for all positions V is represented as below.

$$V = \{\boldsymbol{v_1}, \cdots, \boldsymbol{v_L}\}$$

Also, the number of samples K is limited to a reasonable number of 3 in a single, and 6 in case/control condition at most because most of HTS experiments have been carried out with duplicates or triplicates for each condition.

In addition, let there be read coverage data of K samples for reactIDR, in which the first (latter) $K/2$ samples are treated with nuclease S1 (V1) in PARS dataset, and reagent (DMSO) in icSHAPE dataset, respectively. In that case, $K/2$, represented by k_t hereafter, should be 2 for the experiment with duplicates, and 3 for that with triplicates for each case and control condition. $\boldsymbol{v}_i$ is then defined to consist of read count profiles from case and control samples as

below.

$$\begin{aligned}
\boldsymbol{v}_i &:= \left\{\boldsymbol{v}_{i,case},\ \boldsymbol{v}_{i,cont}\right\} \\
\boldsymbol{v}_{i,case} &= \{v_{i,1}, \cdots, v_{i,k_t}\} \\
\boldsymbol{v}_{i,cont} &= \{v_{i,k_t+1}, \cdots, v_{i,K}\}
\end{aligned}$$

1.2 IDR computation based on Gaussian mixture copula

A basic idea of IDR is to use the mixture copula to discriminate two or greater number of distributions as true and false signals (Figure 4). HTS is performed to produce an enrichment of read distribution at reactive sites in the case condition with higher reproducibility. Hence, the reproducible group is expected to contain more true signals than the irreproducible group coming from a noise such as endogenous fragmentation or stochastic drop off during RT. On the basis of this assumption, IDR applies a Gaussian mixture copula model to extract such reproducible group as a true signal group.

1.2.1 Gaussian copula

Firstly, we describe a copula model of the joint cumulative distribution function for a ranking data that is a vector of length K, referred as $\boldsymbol{u}$. Let $(X_1, X_2, ...X_K)$ be a random vector distributed as multivariate and absolutely continuous distribution with a joint cumulative distribution function g, and marginal cumulative distribution function F_k. Since any cumulative distribution function is within the range $[0, 1]$, there exists x_k, such that $F_k(x_k) = u_k$. This x_k is referred to as a pseudo value of u_k. A copula is a distribution function defined as below:

$$\begin{aligned}
C(u_1, ..., u_K) &= P[U_1 \le u_1, \ldots, U_K \le u_K] \\
&= P[F_1^{-1}(U_1) \le F_1^{-1}(u_1), \ldots, F_K^{-1}(U_K) \le F_K^{-1}(u_K)] \\
&= P[X_1 \le F_1^{-1}(u_1), \ldots, X_K \le F_K^{-1}(u_K)] \\
&= g(x_1, \ldots x_K)
\end{aligned}$$

where $(X_1, X_2, ...X_K)$ are random variables. Then, the joint probability density of $\boldsymbol{u}$ is obtained by the differential of the copula:

$$\begin{aligned}
P(\boldsymbol{u}|\theta) &= \frac{\partial^k C(u_1, \ldots, u_K)}{\partial u_1 \ldots \partial u_K} \\
&= \frac{\partial^K g(F^{-1}(x_1), \ldots F^{-1}(x_K))}{\partial F_1^{-1} \ldots \partial F_K^{-1}} \frac{\partial F_1^{-1}}{\partial u_1} \cdots \frac{\partial F_K^{-1}}{\partial u_K} \\
&= \frac{\partial^K g(F^{-1}(x_1), \ldots F^{-1}(x_K))}{\partial F_1^{-1} \ldots \partial F_K^{-1}} \prod_{k=1}^{K} \frac{1}{f_k(F_k^{-1}(u_k))},
\end{aligned}$$

where f_k is a derivative of F_k with respect to x_k. Among the many functions that satisfy the condition required to be a copula, a Gaussian mixture copula is applied in IDR and reactIDR due to its comprehensiveness and applicability. Although the copula can handle further higher-dimensional correlation, most of HTS were carried out with triplicates or less. Hence, the case of $K = 2$ or $K = 3$ is considered hereafter.

To compute a pseudo value for the case of Gaussian copula, a library of error function (erf) can be used in reactIDR. A cumulative standard distribution can be obtained by erf as follows.

$$
\begin{aligned}
\mathrm{erf}(x) =& \frac{2}{\sqrt{\pi}} \int_0^x e^{-t^2} dt = \frac{2}{\sqrt{2\pi}} \int_0^{x\sqrt{2}} e^{-\frac{p^2}{2}} dp \\
=& 2\left(\frac{1}{\sqrt{2}} \int_{-\infty}^{x\sqrt{2}} e^{-\frac{p^2}{2}} dp - \int_{-\infty}^{0} e^{-\frac{p^2}{2}} dp \right) = \quad 2\left(\Phi(\sqrt{2}x) - \frac{1}{2} \right) \\
(\mathrm{erf}(x))' =& \frac{2}{\sqrt{\pi}} e^{-x^2} \\
\Phi(x) =& \frac{1}{\sqrt{2\pi}} \int_{-\infty}^{x} e^{-t^2} dt = \frac{1}{2}\left(1 + \mathrm{erf}\left(\frac{x}{\sqrt{2}} \right) \right)
\end{aligned}
$$

where $\phi(x)$ and $\Phi(x)$ is a standard normal density and standard normal cumulative distribution function, respectively.

Also, we define a joint cumulative probability function based on two- and three-dimensional Gaussian copula (corresponding to the case of $K = 2$ and $K = 3$) assuming uniform correlation structure.

$$
g(x_1, \ldots x_K) = \int_{-\infty}^{x_1} \cdots \int_{-\infty}^{x_K} f_{(K)}(x'_1, \ldots, x'_K)\, dx'_1 \ldots dx'_K,
$$

where $f_{(K)}$ is a multivariate normal distribution as shown below:

$$
\begin{aligned}
f_{(2)}(\boldsymbol{x}, \boldsymbol{\mu}, \sigma^2, \rho) =& N\left(\left(\begin{array}{c} x_1 \\ x_2 \end{array} \right) \middle| \boldsymbol{\mu}, \left(\begin{array}{cc} \sigma^2 & \sigma^2\rho \\ \sigma^2\rho & \sigma^2 \end{array} \right) \right) \\
=& \frac{1}{2\pi\sqrt{\sigma^4(1-\rho^2)}} \\
& \times \exp\left\{ -\frac{1}{2\sigma^2(1-\rho^2)} (\boldsymbol{x}-\boldsymbol{\mu})^T \left(\begin{array}{cc} 1 & -\rho \\ -\rho & 1 \end{array} \right) (\boldsymbol{x}-\boldsymbol{\mu}) \right\} \\
f_{(3)}(\boldsymbol{x}, \boldsymbol{\mu}, \sigma^2, \rho) =& N\left(\left(\begin{array}{c} x_1 \\ x_2 \\ x_3 \end{array} \right) \middle| \boldsymbol{\mu}, \left(\begin{array}{ccc} \sigma^2 & \sigma^2\rho & \sigma^2\rho \\ \sigma^2\rho & \sigma^2 & \sigma^2\rho \\ \sigma^2\rho & \sigma^2\rho & \sigma^2 \end{array} \right) \right) \\
=& \frac{1}{\sqrt{2\pi}^3\sqrt{\sigma^6(1-\rho)^2(2\rho+1)}} \\
& \times \exp\left\{ -\frac{1}{2\sigma^4(1-\rho)(2\rho+1)} (\boldsymbol{x}-\boldsymbol{\mu})^T \left(\begin{array}{ccc} 1+\rho & \rho & \rho \\ \rho & 1+\rho & \rho \\ \rho & \rho & 1+\rho \end{array} \right) (\boldsymbol{x}-\boldsymbol{\mu}) \right\}
\end{aligned}
$$

where $\boldsymbol{\mu}$, σ^2, and ρ are the parameters to be fitted to an observation $\boldsymbol{u}$. Following [7], we assume spurious signals on the replicates to be independent, that is, $\rho = 0$, while ρ to be positive since true signals are positively correlated, or reproducible among replicates. A marginal cumulative distribution function for each x_k is simply obtained for the Gaussian copula as follows.

$$
F_k(x_k) = \int_{-\infty}^{x_k} N(x'_k|\mu, \sigma^2)\, dx'_k = u_k
$$

1.2.2 Gaussian mixture copula in IDR

The irreproducible discovery rate (IDR) is a method to classify each signal into a true (high-coverage and reproducible) or false (low-coverage and irreproducible) signal based on the Gaussian mixture model by associating observation with the cumulative joint probability distribution and pseudo-value x . For the case of the mixture copula of two (spurious and true) signals assumed in IDR, F_k, g, and a function of the vectorized inverse of F_k F^{-1} are defined as below:

$$
\begin{aligned}
F_k(x_k) &= \int_{-\infty}^{x_k} qN(x'_k|\mu, \sigma^2) + (1-q)N(x'_k|0,1)dx'_k \\
F^{-1}(\boldsymbol{v_j}) &= \{F_1^{-1}(v_{i,j,1}), F_2^{-1}(v_{i,j,1})\} = \boldsymbol{x} \\
g(x_1,\ldots,x_K) &= \int_{-\infty}^{x_1}\cdots\int_{-\infty}^{x_K} qf_{(K)}(\boldsymbol{x},\boldsymbol{\mu},\sigma^2,\rho) + (1-q)f_{(K)}(\boldsymbol{x},\boldsymbol{0},1,0)dx'_1\ldots dx'_K
\end{aligned}
$$

where q corresponds to the ratio of true and false signals in the candidates.

Then, an expected likelihood function Q for the observation of $\boldsymbol{v}_i$ is as follows;

$$
\begin{aligned}
Q(\boldsymbol{\theta}|\boldsymbol{v_i}) &= P(\boldsymbol{v_i}|\ rep,\ \boldsymbol{\theta}) + P(\boldsymbol{v}_i|\ irep,\ \boldsymbol{\theta}) \\
P(\boldsymbol{v}_i|\ rep,\ \boldsymbol{\theta}) &:= \frac{qf_{(K)}(F^{-1}(\boldsymbol{v_i}),\boldsymbol{\mu},\sigma^2,\rho)}{\prod_{k=1}^{K} f_k(F_k^{-1}(v_{i,k}))} \\
P(\boldsymbol{v}_i|\ irep,\ \boldsymbol{\theta}) &:= \frac{(1-q)f_{(K)}(F^{-1}(\boldsymbol{v_i}),\boldsymbol{0},1,0)}{\prod_{k=1}^{K} f_k(F_k^{-1}(v_{i,k}))}
\end{aligned}
\tag{1}
$$

where $P(\boldsymbol{v}_i|\ rep,\ \boldsymbol{\theta})$ and $P(\boldsymbol{v}_i|\ irep,\ \boldsymbol{\theta})$ corresponds to the probability that the ith signal is reproducible and irreproducible, respectively. The third line of Eq. 1 is defined as *local irreproducible discovery rate* (*idr*) in Ref. [7]. The criteria of IDR is defined as follows in analogy to the marginal false discovery rate.

$$
\begin{aligned}
\mathrm{IDR}(\gamma) =& P(\ irep \mid i \in I_\gamma) \\
=& \frac{\int_{I_\gamma}(1-q)f_{(K)}(\boldsymbol{x},\boldsymbol{0},1,0)}{\int_{I_\gamma} qf_{(K)}(\boldsymbol{x},\boldsymbol{\mu},\sigma^2,\rho) + (1-q)f_{(K)}(\boldsymbol{x},\boldsymbol{0},1,0)},
\end{aligned}
$$

where $I_\gamma = \{\boldsymbol{x} : idr(\boldsymbol{x}) < \gamma\}$. Since IDR does not suppose any contextual dependency on each transcript, the product of likelihood function for all positions of each transcript is aimed to be maximized by EM algorithm with pseudo-data.

1.3 Advantage and limitation of IDR

In the previous study, IDR computation was applied to extract reproducible peaks from several sequencing-based analyses such as ChIP-Seq and CAGE-Seq [1, 8]. Although the subject of HTS is different from them, a similar tendency, such as irreproducible read counts observed in low coverage regions, is observed due to the systematic bias of sequencing and library preparation. In addition, IDR has the advantage that it can be estimated on the space of joint distribution of random variables that should be only marginally uniformly distributed between (0, 1) for the copula. This means that IDR evaluation is available for a variety of score distributions, such as read coverages, the ratios of read coverage between different samples, and their p-values, even

though the information on the magnitude of score ranges is discarded by rank transformation in IDR.

To compare the samples in multiple conditions, such as case and control samples of HTS, however, another approach is necessary to utilize IDR concepts effectively because the existing IDR cannot handle the comparison of samples from different conditions. To extend the concept of IDR to that comparison, we developed reactIDR, a novel algorithm designed to extract reproducible true signals from HTS data, including local consistency of IDR as on the HMM. In the following subsection, we describe the detailed algorithm of reactIDR and its optimization process.

1.4 reactIDR

reactIDR is a novel algorithm designed to extract reproducible true signals from HTS, including local consistency of IDR as on the HMM. As summarized in Figure 1 in the main paper, reactIDR receives a wide range of datasets as long as they contain replicates, such as the HTS dataset obtained from multiple conditions. In reactIDR, each latent variables in HMM, except for unmapped class correspond to each status of the RNA secondary structure. Specifically, that enriched in the case and control corresponds to loop- and stem-like (or background-noise) class, respectively, where the case and control samples correspond to $K/2$ samples are nuclease S1 (V1) treated in PARS dataset, and reagent (DMSO) treated in icSHAPE dataset among K experiments. Each latent class is hereafter referred as *unmapped*, *stem*, and *loop*. The set of these three classes is defined as S. Notice that the control samples in probing-based analyses are not necessarily enriched in stem regions, unlike cleavage-based methods. However, there are also several studies that a RT is likely to stop at stable base pairs [9].

In reactIDR, *loop*, *stem*, and *unmapped* classes are estimated to make the enrichment of $v_{i,k}$ in the case, control, and no specific enrichment be observed more frequently at the region of each class, respectively. Therefore, according to the likelihood of being loop or stem from the mixture of reliable and spurious signals, reactIDR classifies each position into class s ($\in S$). When all the parameters in reactIDR are represented by $\boldsymbol{\theta}$, a likelihood of a specific path of latent variables for all nucleotide positions $\boldsymbol{h}$ is formulated as below:

$$P(V, \boldsymbol{h}|\boldsymbol{\theta}) = P(h_0|\boldsymbol{\theta}) \prod_{i=1}^{L} P(\boldsymbol{v_i}|h_i, \boldsymbol{\theta}) \prod_{i=0}^{L-1} P(h_{i+1}|h_i, \boldsymbol{\theta})$$

where h_0 and h_L are always assigned to the *unmapped* class ($P(h_0|\boldsymbol{\theta}) = P(h_L|\boldsymbol{\theta}) = 1$), because sequencing reads are rarely mapped into the region around 5′- and 3′-end. $P(h_{i+1}|h_i, \boldsymbol{\theta})$ and $P(\boldsymbol{v}_i|h_i, \boldsymbol{\theta})$ is a transition probability between h_i and h_{i+1} and an emission probability of $\boldsymbol{v}_i$, respectively (detailed later). Then, a log likelihood ll and Q function Q are obtained as below:

$$\begin{aligned} ll(\boldsymbol{\theta}|D, \boldsymbol{h}) &= \log P(V, \boldsymbol{h}|\boldsymbol{\theta}) \\ Q(\boldsymbol{\theta}|\boldsymbol{\theta}') &= E_{\boldsymbol{h}|V,\boldsymbol{\theta}'}[ll(\boldsymbol{\theta}|D, \boldsymbol{h})] \\ &= E_{\boldsymbol{h}|V,\boldsymbol{\theta}'}[\log P(V, \boldsymbol{h}|\boldsymbol{\theta})] \\ &= \sum_{\boldsymbol{h} \in H} P(\boldsymbol{h}|V, \boldsymbol{\theta}') \log P(V, \boldsymbol{h}|\boldsymbol{\theta}), \end{aligned} \tag{2}$$

where H is the set of possible paths of latent variables and $\boldsymbol{\theta}'$ is an old set of parameters obtained in the previous iteration. The goal of reactIDR is to estimate the parameters maximizing Eq. 2 and optimal latent classes based on their posterior probabilities.

1.4.1 Transition and emission probability

Figure 5 is a schematic illustration of the relationship between reactIDR parameter sets. $\boldsymbol{\theta}$ consists of a transition matrix and two sets of copula parameters $\boldsymbol{\theta_{case}}$ and $\boldsymbol{\theta_{cont}}$, which corresponds to the first and latter half of samples, respectively. More specifically, $\boldsymbol{\theta}$ is defined as follows:

$$\begin{aligned}\boldsymbol{\theta} &= \{\mathcal{R}, \boldsymbol{\theta}_{case}, \boldsymbol{\theta}_{cont}\}\\ \boldsymbol{\theta}_{case} &= \{\boldsymbol{\mu}_1, \sigma_1^2, \rho_1\}\\ \boldsymbol{\theta}_{cont} &= \{\boldsymbol{\mu}_2, \sigma_2^2, \rho_2\}\end{aligned}$$

where $\mathcal{R}$ is a transition matrix between each latent class pair. Among the parameters, $\mathcal{R}$ is mainly required for the transition probability while $\boldsymbol{\theta}_{case}$ and $\boldsymbol{\theta}_{cont}$ are used for emission probability. The emission is defined as follows:

$$\begin{aligned}P(\boldsymbol{v}_i|h_i, \boldsymbol{\theta}) =&P(\boldsymbol{v}_{i,case}|r_{case}, \boldsymbol{\theta}_{case}) \times P(\boldsymbol{v}_{i,cont}|r_{cont}, \boldsymbol{\theta}_{cont})\\ r_{cont} :=&\ \ rep \text{ if } h_i = stem \text{ and } irep \text{ otherwise.}\\ r_{case} :=&\ \ rep \text{ if } h_i = loop \text{ and } irep \text{ otherwise.}\end{aligned} \quad (3)$$

where $\boldsymbol{v}_{case}$ and $\boldsymbol{v}_{cont}$ is expected to show an enrichment of loop-like and stem-like (or background noise) regions, respectively. These probabilities of *rep* and *irep* signals are defined as they are in IDR as follows:

$$\begin{aligned}P(\boldsymbol{v}_{i,j},\ r_j\ |\boldsymbol{\theta}_j) :=&\frac{f_{(k_t)}(F^{-1}(\boldsymbol{v}_{i,j}), \boldsymbol{\mu_h}, \sigma_h^2, \rho_h)}{\prod_{k=1}^{k_t} f_k(F_k^{-1}(v_{i,j,k}))} && \text{if } r_j = rep\\ :=&\frac{f_{(k_t)}(F^{-1}(\boldsymbol{v}_{i,j}), 0, 1, 0)}{\prod_{k=1}^{k_t} f_k(F_k^{-1}(v_{i,j,k}))} && \text{otherwise.}\end{aligned}$$

where $h = 1$ when $j = case$ and $h = 2$ when $j = cont$. In reactIDR, $\boldsymbol{\mu}_j$ is restricted that all elements of $\boldsymbol{\mu}$ are same, as that in IDR. Note that each probability is altered to be $1/q$ or $1/(1-q)$ times as high as the original probability defined in IDR. This is because the influence of the proportion ratio of reproducible and irreproducible signals is considered in the transition probability in reactIDR.

On the other hand, a transition probability is just parametrized by $p(h''|h')$ in $\mathcal{R}$, which satisfies the probability condition:

$$\begin{aligned}P(h''|h', \boldsymbol{\theta}) =&p(h''|h')\\ \sum_{h'} p(h''|h') =&1\end{aligned}$$

In $\mathcal{R}$, the transition probability is altered when the transition occurred from $5'$ to $3'$ and from $3'$ to $5'$, to take into accounts the asymmetrical sequencing efficiency.

1.5 EM algorithm

Using the EM algorithm, each parameter is iteratively optimized to maximize Q function in reactIDR. In E step, a responsibility is computed for the expectation of log likelihood. Then, each parameter is moved to the direction of the derivative of Q function in M step. While the arguments of the maxima are sought in the EM algorithm, the optimization of copula parameters

calls for re-computation of the pseudo-values. Due to the long re-computation time of pseudo-values, reactIDR actually adopts a strategy to set a limitation on the number of parameter optimizations in a single iteration, referred to as generalized EM [10], by fmin_l_bfgs_b library in SciPy.

1.6 E step

In this step, the expectation of log likelihood Q is calculated:

$$\begin{aligned}
Q(\boldsymbol{\theta}|\boldsymbol{\theta}') &= \sum_{\boldsymbol{h}\in H} P(\boldsymbol{h}|V,\boldsymbol{\theta}')\log P(V,\boldsymbol{h}|\boldsymbol{\theta}) \\
&= \sum_{\boldsymbol{h}\in H} P(\boldsymbol{h}|V,\boldsymbol{\theta}')\log\left(P(h_0|\boldsymbol{\theta})\prod_{i=1}^{L} P(\boldsymbol{v}_i|h_i,\boldsymbol{\theta})\prod_{i=0}^{L-1} P(h_{i+1}|h_i,\boldsymbol{\theta})\right) \\
&= \sum_{\boldsymbol{h}\in H} P(\boldsymbol{h}|V,\boldsymbol{\theta}')\sum_{i=1}^{L}\log(P(h_0|\boldsymbol{\theta})) + \log(P(\boldsymbol{v}_i|h_i,\boldsymbol{\theta})) + \log(P(h_{i+1}|h_i,\boldsymbol{\theta})) \\
&= \log P(h_0|\boldsymbol{\theta}) + \sum_{\boldsymbol{h}\in H}\sum_{i=1}^{L} P(\boldsymbol{h}|V,\boldsymbol{\theta}')\log(P(\boldsymbol{v}_i|h_i,\boldsymbol{\theta})) + \sum_{\boldsymbol{h}\in H}\sum_{i=0}^{L-1} P(\boldsymbol{h}|V,\boldsymbol{\theta}')\log(P(h_{i+1}|h_i,\boldsymbol{\theta}))
\end{aligned}$$

Of the three terms in the above formula, the first term is 0. The second and third term are obtained as follow:

$$\sum_{\boldsymbol{h}\in H}\sum_{i=1}^{L} P(\boldsymbol{h}|V,\boldsymbol{\theta}')\log(P(\boldsymbol{v}_i|h_i,\boldsymbol{\theta})) = \sum_{i=1}^{L}\sum_{h'\in S}\gamma(h'_i|V,\boldsymbol{\theta}')\log(P(\boldsymbol{v_i}|h'_i,\boldsymbol{\theta}))$$

$$\sum_{\boldsymbol{h}\in H}\sum_{i=0}^{L-1} P(\boldsymbol{h}|V,\boldsymbol{\theta}')\log(P(h_{i+1}|h_i,\boldsymbol{\theta})) = \sum_{h',h''\in S}\gamma(h',h''|V,\boldsymbol{\theta}')\log(P(h''|h',\boldsymbol{\theta})),$$

where a responsibility γ is defined for each position and transition state as below:

$$\gamma(h'_i|V,\boldsymbol{\theta}') := \sum_{\boldsymbol{h}\in H} P(\boldsymbol{h}|V,\boldsymbol{\theta}')I(h_i = h'_i)$$

$$\gamma(h',h''|V,\boldsymbol{\theta}') := \sum_{\boldsymbol{h}\in H}\sum_{j=0}^{L-1} P(\boldsymbol{h}|V,\boldsymbol{\theta}')I(h_j = h')I(h_{j+1} = h''),$$

where I is an indicator function. These responsibilities can be obtained by forward-backward algorithm effectively as follows.

$$f(0,h') = \begin{cases} 1 & \text{if } h' = \textit{unmapped}, \\ 0 & \text{otherwise.} \end{cases}$$

$$f(i,h') = \sum_{h''\in S} f(i-1,h'')P(h'|h'',\boldsymbol{\theta})P(\boldsymbol{v_i}|h',\boldsymbol{\theta})$$

$$f(L,h') = \sum_{h''\in S} f(L-1,h'')P(h'|h'',\boldsymbol{\theta})$$

$$b(L, h') = \begin{cases} 1 & \text{if } h' = unmapped, \\ 0 & \text{otherwise.} \end{cases}$$

$$b(i, h') = \sum_{h'' \in S} b(i+1, h'') P(h'' | h', \boldsymbol{\theta}) P(\boldsymbol{v_i} | h', \boldsymbol{\theta})$$

$$b(0, h') = \sum_{h'' \in S} b(1, h'') P(h'' | h', \boldsymbol{\theta})$$

Then, a responsibility is obtained as below.

$$\gamma(h'_i | V, \boldsymbol{\theta}') := \sum_{\boldsymbol{h}'' \in S} f(i-1, h'_i) P(h'' | h'_i, \boldsymbol{\theta}) b(i, h'')$$

$$\gamma(h', h'' | V, \boldsymbol{\theta}') := \sum_{\boldsymbol{h} \in H} \sum_{i=0}^{L-1} f(i, h_i) P(h_{i+1} | h_i, \boldsymbol{\theta}) b(i+1, h_{i+1}) I(h_i = h') I(h_{i+1} = h''),$$

1.7 M step

In this step, a new optimal $\boldsymbol{\theta}$ is obtained to maximize the expectation of log likelihood, resulting in fitting the model to the observation. The new $\boldsymbol{\theta}$ should satisfy the equation $\boldsymbol{\theta} = \arg\max_{\boldsymbol{\theta}} Q(\boldsymbol{\theta} | \boldsymbol{\theta}')$ in EM. The posterior for an old parameter $\boldsymbol{\theta}'$, such as $\gamma(h'_i | V, \boldsymbol{\theta}')$ and $\gamma(h', h'' | V, \boldsymbol{\theta}')$, has been already computed in the previous step.

1.7.1 M step: optimization of the transition matrix for transition probability

To obtain the optimal solution of transition probability $p(h''|h')$, Q function is differentiated by $p(h''|h')$, considering the constraint that the sum of $p(h''|h')$ is equal to one by introducing a Lagrange multiplier:

$$\begin{aligned}
\frac{\partial Q}{\partial p(h''|h')} &= \frac{\partial}{\partial p(h''|h')} \left\{ \sum_{\boldsymbol{h} \in H} \sum_{i=0}^{L-1} P(\boldsymbol{h} | V, \boldsymbol{\theta}') \log P(h_{i+1} | h_i, \boldsymbol{\theta}') + \lambda \left(\sum_{h''} p(h''|h') - 1 \right) \right\} \\
&= \frac{\partial}{\partial p(h''|h')} \left\{ \gamma(h', h'' | V, \boldsymbol{\theta}') \log p(h''|h') \right\} + \lambda \\
&= \sum_{h \in H} \sum_{i=0}^{L-1} \gamma(h', h'' | V, \boldsymbol{\theta}') / p(h''|h') + \lambda \\
&= N(h', h'' | \boldsymbol{\theta}') / p(h''|h') + \lambda = 0,
\end{aligned}$$

where $N(h', h''|\boldsymbol{\theta}')$ is an expectation count of transition between h' and h'', and λ is a Lagrange multipler. This leads to the following equation regarding λ:

$$\lambda = N(h', h'' | \boldsymbol{\theta}') / p(h''|h')$$

Because λ is an only variable, the relationship between $p(h''|h')$ for any h' and h'' is derived as follows:

$$\begin{aligned}
p(h'''|h') =& \frac{N(h', h''')}{N(h', h'')} p(h''|h') \\
\sum_{h''' \in S} p(h'''|h') =& \frac{\sum_{h'''} N(h', h''' | \boldsymbol{\theta}')}{N(h', h'' | \boldsymbol{\theta}')} p(h''|h') = 1 \\
p(h''|h') =& \frac{N(h', h'' | \boldsymbol{\theta}')}{\sum_{h'''} N(h', h''' | \boldsymbol{\theta}')}
\end{aligned}$$

Thus, an optimal solution of transition probability is simply the expectation ratio of transition between each state. Then, we define a transition matrix $\mathcal{R}$ that consists of these transition probabilities p.

In the HMM model, a parameter of reproducible peaks, q (π in the original paper) should correspond to the ratio of latent variables. In this model, therefore, q_{loop} and q_{stem} are estimated after updating $\mathcal{R}$. When $\mathcal{R}$ satisfies the probability condition for each row, it can be converted into the orthogonal matrix of eigenvalues D by a matrix P:

$$\mathcal{R}P = \mathcal{R}(\boldsymbol{x_1}, \boldsymbol{x_2}, \boldsymbol{x_3}) = (\lambda_1\boldsymbol{x_1}, \lambda_2\boldsymbol{x_2}, \lambda_3\boldsymbol{x_3})$$
$$P^{-1}\mathcal{R}P = D = \begin{pmatrix} \lambda_1 & 0 & 0 \\ 0 & \lambda_2 & 0 \\ 0 & 0 & \lambda_3 \end{pmatrix},$$

where $\boldsymbol{x_1}, \boldsymbol{x_2}, \boldsymbol{x_3}$ are eigenvectors, and $\lambda_1, \lambda_2, \lambda_3$ are eigenvalues of $\mathcal{R}$. The expectation vector of the duration time $\boldsymbol{d}$ ($d_{unmapped}, d_{loop}, d_{stem}$) for V is obtained as follows:

$$\begin{aligned} E[\boldsymbol{d}] &= \mathcal{R} + \mathcal{R}^2 + \cdots + \mathcal{R}^L \\ &= \frac{1}{L}\left(PDP^{-1} + PD^2P^{-1} + \cdots + PD^LP^{-1}\right) \\ &= \frac{1}{L}P\begin{pmatrix} a(\lambda_1) & 0 & 0 \\ 0 & a(\lambda_2) & 0 \\ 0 & 0 & a(\lambda_3) \end{pmatrix}P^{-1} \quad \left(a(\lambda) := \frac{\lambda(1-\lambda^L)}{1-\lambda}\right) \end{aligned}$$

When the sequence length L is so long that the expectation of duration time can be approximated by the equilibrium distribution, it is obtained by the eigenvector $\boldsymbol{d}$, which corresponds to the largest eigenvalue of one. Specifically, the ratios of reproducible samples for *stem* and *loop* are obtained as follows, respectively:

$$\begin{aligned} q_{stem} &= d_{stem}/|\boldsymbol{d}| \\ q_{loop} &= d_{loop}/|\boldsymbol{d}| \end{aligned}$$

where q_{stem} and q_{loop} are always obtained to be $1/3$.

1.7.2 M step: optimization of the copula parameters for emission probability

Since there is assumed to be no dependency between the reproducibility strength of case and control sample, reactIDR individually optimizes the parameters for the mixture copula model $\boldsymbol{\mu}$, σ, and ρ a for each sample ($\boldsymbol{\theta_{case}}$ and $\boldsymbol{\theta_{cont}}$). A derivative of Q in terms of copula parameters θ_j is as below.

$$\begin{aligned} \frac{\partial Q}{\partial \boldsymbol{\theta_j}} &= \left(\sum_{i=1}^{L}\sum_{h'\in S}\gamma(h'_i|V,\boldsymbol{\theta'})\log(P(\boldsymbol{v}_i|h_i,\boldsymbol{\theta}))\right)' \\ &= \sum_{i=1}^{L}\sum_{h'_i\in S}\gamma(h'_i|V,\boldsymbol{\theta'})\frac{\partial\log(P(\boldsymbol{v}_{case}|h'_i,\boldsymbol{\theta}_j))}{\partial\boldsymbol{\theta_j}} \end{aligned}$$

We describe the derivative of Q for some position i because Q (and its derivative) can be obtained by the sum of Q for each position.

Let define $\boldsymbol{v_{i,case}}$ as $\boldsymbol{u}_{case}$, $\boldsymbol{v}_{i,cont}$ as $\boldsymbol{u}_{cont}$, and h_i as h' for simplicity. In the logarithm form, a derivative of $P(\boldsymbol{u}_j|h', \boldsymbol{\theta_j})$ (defined in Eq. 3) by $\boldsymbol{\theta_j}$ can be rewritten as follows:

$$\frac{\partial \log P(\boldsymbol{u}_j|h', \boldsymbol{\theta_j})}{\partial \boldsymbol{\theta_j}} = \log(P(\boldsymbol{u_j}|\ r_j, \boldsymbol{\theta_j}))'$$
$$= \begin{cases} (\log(R(\boldsymbol{u_j}, \boldsymbol{\theta_j}) - \log(S(\boldsymbol{u_j}, \boldsymbol{\theta_j}))' & \text{if } r_j = rep \\ (\log(I(\boldsymbol{u_j}, \boldsymbol{\theta_j}) - \log(S(\boldsymbol{u_j}, \boldsymbol{\theta_j}))' & \text{otherwise.} \end{cases}$$

R, I, and S are defined as follows

$$R(\boldsymbol{u_j}, \boldsymbol{\theta_j}) = f_{(k_t)}(\boldsymbol{x}|\boldsymbol{\mu_h}, \sigma_h^2, \rho_h)$$
$$I(\boldsymbol{u_j}, \boldsymbol{\theta_j}) = f_{(k_t)}(\boldsymbol{x}|\boldsymbol{0}, 1, 0)$$
$$S(\boldsymbol{u_j}, \boldsymbol{\theta_j}) = \prod_{k=1}^{k_t} f_k(x_k|\boldsymbol{\theta_j})$$
$$x_k = F_k^{-1}(u_{jk}) = \Phi^{-1}(u_{jk})$$

where $h = 1$ when $j = case$ and $h = 2$ when $j = cont$, and $\Phi(x)$ is a standard normal cumulative distribution function. Since each F_k^{-1} also depends on $\boldsymbol{\theta}$, a derivative of $\log P(\boldsymbol{u}_j|h', \boldsymbol{\theta_j})$ is obtained as follows:

$$\frac{\partial \log P(\boldsymbol{u}_j|h', \boldsymbol{\theta_j})}{\partial \boldsymbol{\theta_j}} = \frac{\partial \log P(\boldsymbol{u}_j|h', \boldsymbol{\theta_j})}{\partial \boldsymbol{\theta_j}}(F_1^{-1}(u_{j1}), \ldots, F_{k_t}^{-1}(u_{j,k_t})) + \sum_{k=1}^{k_t} \frac{\partial F_k^{-1}(u_{jk})}{\partial \boldsymbol{\theta_j}} \frac{\partial \log P(\boldsymbol{u}_j|h', \boldsymbol{\theta_j})}{\partial x_k} \tag{4}$$

Therefore, three variables appeapred in Eq. 4 are required to compute the derivative of emission probability with respect to each copula parameter θ.

For example, the general case of $k_t = 2$ is obtained following Taylor expansion and chain rule as follows.

$$\frac{df_\theta(F_{1,\theta}^{-1}(u_{j,1}), F_{2,\theta}^{-1}(u_{j,2}))}{d\theta} = \lim_{\Delta\theta \to 0} \frac{f_{\theta+\Delta\theta}(F_{1,\theta+\Delta\theta}^{-1}(u_{j,1}), F_{2,\theta+\Delta\theta}^{-1}(u_{j,2})) - f_\theta(F_{1,\theta}^{-1}(u_{j,1}), F_{2,\theta}^{-1}(u_{j,2}))}{\Delta\theta}$$
$$= \lim_{\Delta\theta \to 0} \frac{f_{\theta+\Delta\theta}(F_{1,\theta}^{-1}(u_{j,1}) + \Delta\theta\frac{\partial F_1^{-1}}{\partial\theta}, F_{2,\theta}^{-1}(u_{j,2}) + \Delta\theta\frac{\partial F_2^{-1}}{\partial\theta}) - f_\theta(F_{1,\theta}^{-1}(u_{j,1}), F_{2,\theta}^{-1}(u_{j,2}))}{\Delta\theta}$$
$$= \lim_{\Delta\theta \to 0} \frac{\partial f(F_{1,\theta}^{-1}(u_{j,1}), F_{2,\theta}^{-1}(u_{j,2}))}{\partial\theta} + \frac{\partial F_1^{-1}(u_{j,1})}{\partial\theta}\frac{\partial f}{\partial x_1} + \frac{\partial F_2^{-1}(u_{j,2})}{\partial\theta}\frac{\partial f}{\partial x_2} + O(\Delta\theta)$$
$$= \frac{\partial f}{\partial\theta}(F_{1,\theta}^{-1}(u_{j,1}), F_{2,\theta}^{-1}(u_{j,2})) + \frac{\partial F_1^{-1}(u_{j,1})}{\partial\theta}\frac{\partial f}{\partial x_1} + \frac{\partial F_2^{-1}(u_{j,2})}{\partial\theta}\frac{\partial f}{\partial x_2}$$

where f corresponds to $\log P(\boldsymbol{u}_j|h', \boldsymbol{\theta_j})$ in reactIDR.

1.7.3 Case of two-dimensional mixture copula

In this subsection, we will explain how to obtain each term in Eq. 4 for a two-dimensional mixture copula, in which $K = 4$ and $k_t = 2$. For simplicity, we omit the subscript not only i but also j and h from the parameters. Thus, $\boldsymbol{u}$, $\boldsymbol{\theta}$, r, $\boldsymbol{\mu}$, σ^2, ρ, and q correspond to $\boldsymbol{u}_{case}$, r_{case}, $\boldsymbol{\theta}_{case}$, $\boldsymbol{\mu}_1$, σ_1^2, ρ_1, and q_{loop} for the case samples, and $\boldsymbol{u}_{cont}$, r_{cont}, $\boldsymbol{\theta}_{cont}$, $\boldsymbol{\mu}_2$, σ_2^2, ρ_2, and q_{loop} for

the control samples. It is because a derivation of Eq. 4 can be carried out in the same way for the case of $j = case$ and $j = cont$. In addition, all elements of $\boldsymbol{\mu}$ are set to μ, as those defined in IDR. We describe the way to compute a derivative of Q by Eq. 4 following the order shown below.

1. Compute $\frac{\partial F_k^{-1}(u_k)}{\partial \theta}$
   1.1 Compute $\frac{\partial F_k(F_k^{-1}(u_k))}{\partial \theta}$
2. $\frac{\partial \log P(\boldsymbol{u}|h',\boldsymbol{\theta})}{\partial x_k}$
   2.1 Compute $\frac{\partial R}{\partial x_k}$
   2.2 Compute $\frac{\partial I}{\partial x_k}$
   2.3 Compute $\frac{\partial S}{\partial x_k}$
3. Compute $\frac{\partial \log P(\boldsymbol{u}|h',\boldsymbol{\theta})}{\partial \theta}$
   3.1 Compute $\frac{\partial R}{\partial \theta}$
   3.2 Compute $\frac{\partial I}{\partial \theta}$
   3.3 Compute $\frac{\partial S}{\partial \theta}$

Firstly, the step 1, or computing $\frac{\partial F_k^{-1}(u_k)}{\partial \theta}$ is done as follows.

$$\frac{\partial F_k^{-1}(u_k)}{\partial \theta} = -\frac{\frac{\partial F_{k,\theta}}{\partial \theta}(F_k^{-1}(u_k))}{\frac{\partial F_k}{\partial x_k}} = -\frac{\frac{\partial F_k \theta}{\partial \theta}(F_k^{-1}(u_k))}{f_k(x_k)}$$

where $\frac{\partial F_k^{-1}(u_k)}{\partial \theta}$ $\left(\frac{\partial x_k}{\partial \theta}\right)$ can be calculated as follows:

$$\begin{aligned} F_k(F_k^{-1}(u_k)) &= u_k \\ \frac{\partial F_k(F_k^{-1}(u_k))}{\partial \theta} &= \frac{\partial F_k}{\partial \theta} F_k^{-1} + \frac{\partial F^{-1}}{\partial \theta} F_k = 0 \end{aligned}$$

The numerator of the above equation can be formulated for each parameter, μ, σ^2, and ρ in the following ways (as the step 1.1).

$$\begin{aligned} \frac{\partial F_k}{\partial \mu}(F_k^{-1}(u_k)) &= \left(q\Phi\left(\frac{x_k - \mu}{\sqrt{\sigma^2}}\right) + (1-q)\Phi(x_k)\right)' \\ &= -qN(x_k|\mu, \sigma^2) \\ \frac{\partial F_k}{\partial \sigma^2}(F_k^{-1}(u_k)) &= -\left(\frac{x_k - \mu_k}{2\sigma^2}\right) qN(x_k|\mu, \sigma^2) \\ \frac{\partial F_k}{\partial \rho}(F_k^{-1}(u_k)) &= 0 \end{aligned}$$

Next, the derivative of emission probability with respect to x_1 can be obtained as follows (as the step 2):

$$\frac{\partial \log P(\boldsymbol{u}|h', \boldsymbol{\theta})}{\partial x_1} = \begin{cases} (\log R - \log S)' & \text{if } r = rep \\ (\log I - \log S)' & \text{otherwise.} \end{cases}$$

When there are two replicates for two conditions, several variables required for the derivative of emission probabilities are formulated as below:

$$
\begin{aligned}
\frac{\partial}{\partial x_1}\log R &= -\frac{(x_1-\mu)-\rho(x_2-\mu)}{\sigma^2(1-\rho^2)} \\
\frac{\partial}{\partial x_1}\log I &= -x_1 \\
\frac{\partial}{\partial x_1}\log S &= (\log S_1 + \log S_2)' \\
&= -\left(\frac{\frac{(x_1-\mu)}{\sigma^2} qN(x_1|\mu,\sigma^2) + x_1(1-q)N(x_1|0,1))}{S_1}\right)
\end{aligned}
$$

The derivative of emission probability with respect to x_2 can be easily obtained by exchanging x_1 and x_2, and S_1 and S_2.

Lastly, the derivatives of $\frac{\partial \log P(\boldsymbol{v_i}|h_i,\boldsymbol{\theta})}{\partial\theta}$ with respect to a copula parameter is obtained as shown below (as the step 3).

$$
\frac{\partial \log P(\boldsymbol{u}|h',\boldsymbol{\theta})}{\partial\theta}(x_1,\cdots,x_{k_t}) = \begin{cases} (\log R - \log S)' & \text{if } r = rep \\ (\log I - \log S)' & \text{otherwise.} \end{cases}
$$

Let define s as below.

$$
s = (x_1-\mu)^2 + (x_2-\mu)^2 - 2\rho(x_1-\mu)(x_2-\mu)
$$

Then, a derivation with respect to μ is formulated as follows.

$$
\begin{aligned}
\frac{\partial \log R}{\partial\mu} &= \left(\log N\left(\begin{array}{c} x_1 \\ x_2 \end{array}\right)\middle|\left(\begin{array}{c} \mu \\ \mu \end{array}\right), \left(\begin{array}{cc} \sigma^2 & \sigma^2\rho \\ \sigma^2\rho & \sigma^2 \end{array}\right)\right)' \\
&= \left(-\frac{s}{2\sigma^2(1-\rho^2)}\right)' \\
&= \frac{(x_1-\mu)+(x_2-\mu)+\rho(2\mu-(x_1+x_2))}{\sigma^2(1-\rho^2)} \\
&= \frac{(x_1+x_2-2\mu)}{\sigma^2(1+\rho)} \\
\frac{\partial \log I}{\partial\mu} &= 0 \text{ (const)} \\
\frac{\partial \log S}{\partial\mu} &= \sum_{k=1}^{2} \log(S_i)' \\
&= \sum_{k=1}^{2} \frac{qN(x_k|\mu,\sigma^2)}{S_k}\left(\frac{x_i-\mu}{\sigma^2}\right)
\end{aligned}
$$

A derivation with respect to σ^2 is formulated as follows.

$$\begin{aligned}
\frac{\partial \log R}{\partial \sigma^2} &= \left(-\frac{\log \sigma^2}{2} - \frac{s}{2\sigma^2(1-\rho^2)}\right)' \\
&= -\frac{1}{2\sigma^2} + \frac{s}{2\sigma^4(1-\rho^2)} \\
\frac{\partial \log I}{\partial \sigma^2} &= 0 \text{ (const)} \\
\frac{\partial \log S}{\partial \sigma^2} &= \sum_{k=1}^{2} \log(S_i)' \\
&= \sum_{k=1}^{2} \frac{qN(x_k|\mu,\sigma^2)}{S_k}\left(-\frac{1}{2\sigma^2} + \frac{(x_k-\mu)^2}{2\sigma^4}\right)
\end{aligned}$$

A derivation with respect to ρ is formulated as follows.

$$\begin{aligned}
\frac{\partial \log R}{\partial \rho} &= \left(-\frac{\log(1-\rho^2)}{2} - \frac{s}{2\sigma^2(1-\rho^2)}\right)' \\
&= \frac{\rho}{1-\rho^2} + \frac{(x_1-\mu)(x_2-\mu)(1-\rho^2) - \rho s}{\sigma^2(1-\rho^2)^2} \\
\frac{\partial \log I}{\partial \rho} &= 0 \text{ (const)} \\
\frac{\partial \log S}{\partial \rho} &= 0 \text{ (const)}
\end{aligned}$$

1.7.4 Case of three-dimensional mixture copula

In this subsection, we will explain how to obtain each term in Eq. 4 for a three-dimensional mixture copula, in which $K = 6$ and $k_t = 3$. When there are three replicates, several variables required for the derivative of emission probabilities are formulated as below:

$$\begin{aligned}
s = &((x_1-\mu)^2 + (x_2-\mu)^2 + (x_3-\mu)^2)(1+\rho) \\
&- 2\rho((x_1-\mu)(x_2-\mu) + (x_2-\mu)(x_3-\mu) + (x_3-\mu)(x_1-\mu))
\end{aligned}$$

In a similar way to the case of two-dimensional copula, the derivatives of R, I, and S with respect to x_1 are obtained as follows.

$$\begin{aligned}
\frac{\partial}{\partial x_1}\log R &= -\frac{(x_1-\mu)(1+\rho) - \rho((x_2-\mu)+(x_3-\mu))}{\sigma^2(1-\rho)(2\rho+1)} \\
\frac{\partial}{\partial x_1}\log I &= -x_1 \\
\frac{\partial}{\partial x_1}\log S &= (\log S_1 + \log S_2 + \log S_3)' \\
&= -\left(\frac{\frac{(x_1-\mu)}{\sigma^2} qN(x_1|\mu,\sigma^2) + x_1(1-q)N(x_1|0,1)}{S_1}\right)
\end{aligned}$$

The derivative of emission probability with respect to x_k can be easily obtained by replacing x_1 and x_k, and S_1 and S_k.

The derivatives of R, I, and S with respect to each copula parameter are obtained as shown below. A derivation with respect to μ is formulated as follows.

$$\begin{aligned}
\frac{\partial \log R}{\partial \mu} &= \left(\log N\left(\left(\begin{array}{c} x_1 \\ x_2 \\ x_3 \end{array}\right) \middle\| \left(\begin{array}{c} \mu \\ \mu \\ \mu \end{array}\right), \left(\begin{array}{ccc} \sigma^2 & \sigma^2\rho & \sigma^2\rho \\ \sigma^2\rho & \sigma^2 & \sigma^2\rho \\ \sigma^2\rho & \sigma^2\rho & \sigma^2 \end{array}\right)\right)\right)' \\
&= \left(-\frac{s}{2\sigma^2(1-\rho)(2\rho+1)}\right)' \\
&= \frac{\sum_{k=1}^{K}(x_k-\mu)(1+\rho) + 2\rho\sum_{i=1}^{3}\sum_{j\neq i}(2\mu-(x_i+x_j))}{\sigma^2(1-\rho)(2\rho+1)} \\
&= -\frac{3\mu-(x_1+x_2+x_3)}{\sigma^2(2\rho+1)} \\
\frac{\partial \log I}{\partial \mu} &= 0 \text{ (const)} \\
\frac{\partial \log S}{\partial \mu} &= \sum_{k=1}^{3} \log(S_i)' \\
&= \sum_{k=1}^{3} \frac{qN(x_k|\mu,\sigma^2)}{S_k}\left(\frac{x_i-\mu}{\sigma^2}\right)
\end{aligned}$$

A derivation with respect to σ^2 is formulated as follows.

$$\begin{aligned}
\frac{\partial \log R}{\partial \sigma^2} &= \left(-\frac{3\log\sigma^2}{2} - \frac{s}{2\sigma^2(1-\rho)(2\rho+1)}\right)' \\
&= -\frac{3}{2\sigma^2} + \frac{s}{2\sigma^4(1-\rho)(2\rho+1)} \\
\frac{\partial \log I}{\partial \sigma^2} &= 0 \text{ (const)} \\
\frac{\partial \log S}{\partial \sigma^2} &= \sum_{k=1}^{2} \log(S_i)' \\
&= \sum_{k=1}^{3} \frac{qN(x_k|\mu,\sigma^2)}{S_k}\left(-\frac{1}{2\sigma^2} + \frac{(x_k-\mu)^2}{2\sigma^4}\right)
\end{aligned}$$

A derivation with respect to ρ is formulated as follows.

$$
\begin{aligned}
\frac{\partial \log R}{\partial \rho} &= \left(-\frac{\log(1-\rho)^2(2\rho+1)}{2} - \frac{s}{2\sigma^2(1-\rho)(2\rho+1)}\right)' \\
&= -\frac{6\rho^2-6\rho}{(1-\rho)^2(2\rho+1)} - \frac{s'(1-\rho)(2\rho+1)-(-4\rho+1)s}{2\sigma^2(1-\rho)^2(2\rho+1)^2} \\
&= \frac{3\rho}{(1-\rho)(2\rho+1)} - \frac{\sum_{k=1}^{3}(x_k-\mu)^2((1-\rho)(2\rho+1)-(1-4\rho)(1+\rho))}{2\sigma^2(1-\rho^2)(2\rho+1)} \\
&\quad + \frac{2\sum_{i=1}^{3}\sum_{j\neq i}(x_i-\mu)(x_j-\mu)((1-\rho)(2\rho+1)-(1-4\rho)\rho)}{2\sigma^2(1-\rho)^2(2\rho+1)^2} \\
&= \frac{3\rho}{(1-\rho)(2\rho+1)} - \frac{\sum_{k=1}^{3}(x_k-\mu)^2(\rho(2+\rho)) - \sum_{i=1}^{3}\sum_{j\neq i}(x_i-\mu)(x_j-\mu)(2\rho^2+1)}{\sigma^2(1-\rho)^2(2\rho+1)^2} \\
\frac{\partial \log I}{\partial \rho} &= 0 \text{ (const)} \\
\frac{\partial \log S}{\partial \rho} &= 0 \text{ (const)}
\end{aligned}
$$

1.8 Implementation and setting for supervised learning

reactIDR has been implemented in Python 3.x with Cython and publicly available at `https://github.com/carushi/reactIDR` with the GPL v2.0 license, which is based on the previous IDR python implementation appeared at `https://github.com/nboley/idr`. In reactIDR, duplicates or triplicates of HTS data are allowed as the input. To reduce a computational time and avoid an overflow for a genome-wide dataset, reactIDR has equipped two options of multiprocess mode and independent HMM. With the independent HMM, reactIDR is able to do perform "independent" HMM computation, in which a forward-backward algorithm is applied to each transcript while an optimization of parameters is applied for all transcripts at once. The second option enables reactIDR to do multiprocess computation during time-consuming steps such as pseudo-value computation. In addition, the simplest way of genome-wide reactIDR analysis by independently computing IDR for each transcript is enabled with "each" option.

We also provide a docker image at `https://hub.docker.com/r/carushi/rt_end_counter/` to obtain an input of reactIDR in the appropriate format from the bam file of HTS data. In the docker image, the number of the sequencing reads that start at the subsequent (3′) base is counted and written into a bed file, considering the quality, soft-clipped bases, and the maximum length of sequencing reads. A detailed manual is available at `https://github.com/carushi/reactIDR`.

In this study, we applied reactIDR to the problem of structure prediction for rRNAs. We firstly optimized the parameters of reactIDR by fixing the latent class to the reference structure of 18S rRNA, then the structure score was predicted for target rRNAs. At that time, a training and testing was carried out with the negative data for the minus strand of each rRNA.

1.9 Suitability of reactIDR to high-throughput structural analyses

To evaluate the reproducibility of high-throughput sequencing data, assessing the quality of data is widely performed by evaluating how the top-ranked signals are reproducible through a certain correlation coefficient such as Pearson's or Spearman's correlation coefficient. However, that index is occasionally not suitable for measuring the reproducibility, particularly when

measuring the data from the heterogeneous experiments, distribution of arbitrary scores (*e.g.*, reactivity score), or that with missing values [1, 11]. For example, the severe defect of Pearson's correlation coefficient is a susceptibility to outliers, which exist in varied ranges depending on the choice of scoring scheme. On the other hand, the Spearman's correlation is the nonparametric measure for the rank values of two variables. Due to the rank transformation, the outliers may be less influential on the Spearman's correlation coefficient even though the information on the magnitude of score ranges is discarded. The Spearman's correlation coefficient for high-throughput sequencing data, however, still highly depends on the threshold to filter out the lower-ranked samples to evaluate meaningful reproducibility as well as the difficult setting of a single threshold to multiple experiments. In the IDR paper, the authors describe the reproducibility as the extent to which the ChIP-Seq peak ranks are no longer consistent across replicates in decreasing certain significance. By automatic classification into true or spurious signals based on the Gaussian mixture copula, IDR can quantify the reproducibility only from true(-ish) signals depending on the consistency of the samples at each rank range without an arbitrary threshold for each distribution. Moreover, the sequencing reads obtained from HTS analyses show a peak-like distribution similar to that of ChIP-Seq, where IDR is originally intended to apply. Taken together, the existing IDR method and reactIDR can be considered theoretically applicable to the HTS analyses.

In this study, we applied reactIDR to handle both types of PARS-like (PARS) and SHAPE-like (icSHAPE) methods. For efficient optimization in a supervised learning, the enrichment of truncated reads from case and control samples were associated with the *loop* and *stem* class respectively. The control samples of icSHAPE correspond to the condition without any chemical treatment while those of PARS are S1 treated, respectively, in which the control samples do not correspond to literally control samples. A latent class *stem* is, thus, supposed to be a position of background (*bg*) noise in icSHAPE control samples as described in the main paper, but reactIDR assigns *stem* to be base-paired during supervised learning for efficient parameter optimization. This is because reactIDR assumes control samples as the subject competing with case samples. For the characteristics of this basis, reactIDR utilizes *stem* class in order to remove false positives from SHAPE-like methods while stem structures are detected by PARS-like methods. Additionally, there is an issue of no original *unmapped* locus in a supervised learning. Thus, we used the complementary transcripts of each rRNA and the read count distribution aligned to them as the next best. If there is a prior knowledge of *unmapped* region such as spike-in or negative controls, reactIDR can optimize its parameters based on that information.

2 Data processing

2.1 PARS score computation

PARS data consist of two kinds of sequenced samples; one treated with S1 nuclease to measure accessibility, and another treated with RNase V1 to measure the structure stability at each position [12]. To calculate the PARS score, a raw read depth is first normalized with regards to the number of total mapped reads for each condition (represented as $c_{\text{PARS\_condition}}(i,t)$) [12]. In the first PARS study, PARS score is calculated as below:

$$\text{PARS score}(i,t) = \log_2\left(\frac{c_{\text{PARS\_V1}}(i,t)+1}{c_{\text{PARS\_S1}}(i,t)+1}\right),$$

where $c_{\text{PARS\_V1}}(i,t)$ and $c_{\text{PARS\_S1}}(i,t)$ correspond to the normalized read coverage of a V1 (S1) dataset at the ith nucleotide (detailed in Ref. [12]). When replicates are available, $c_{\text{PARS\_V1}}$ and $c_{\text{PARS\_S1}}$ are obtained using an average or sum of read counts. The PARS score is then capped to ±7 for scaling.

In Ref. [13], however, the PARS score is redefined as a five-base average of the previous PARS scores, as below:

$$\text{PARS score}(i,t) = \log_2\left(\sum_{j=i-2}^{i+2} \frac{c_{\text{PARS\_V1}}(j,t)+5}{5}\right) - \log_2\left(\sum_{j=i-2}^{i+2} \frac{c_{\text{PARS\_S1}}(j,t)+5}{5}\right)$$

In this study, the latter PARS score r_{PARS} was computed using the mean of $c_{\text{PARS\_V1}}$ and $c_{\text{PARS\_S1}}$ to compute a single score from replicates. This is because such averaging can buffer local ambiguity of read mapping in exchange for the detailed resolution. Actually, the consistency of PARS with computational prediction was already demonstrated in ParasoR research [14].

2.2 icSHAPE score computation

Another method, icSHAPE [15, 16], detects flexible nucleotides using NAI-N_3 reagent by observing the enrichment of RT drop-off compared to the control DMSO sample. In [16], a reactivity score $r_{\text{icSHAPE\_condition}}(i,t)$ is defined as below.

$$\text{icSHAPE score}(i,t) = \\ (c_{\text{icSHAPE\_condition}}(i,t) - \alpha c_{\text{icSHAPE\_DMSO}}(i,t))/c_{\text{icSHAPE\_background, DMSO}}(i,t),$$

where $c_{\text{icSHAPE\_condition}}$ is a normalized read count, $c_{\text{icSHAPE\_background, DMSO}}(i,t)$ is the number of read counts that read through the ith nucleotide, and α is a parameter to adjust the strength of background control and set it to 0.25 in this study, as optimized in the previous study [15]. $c_{\text{icSHAPE\_condition}}$ and $c_{\text{icSHAPE\_DMSO}}$ is the normalized read coverage, or the ratio of RT stop read counts against the whole sequencing library. At this time, $c_{\text{icSHAPE\_condition}}$ and $c_{\text{icSHAPE\_DMSO}}$ are scaled so that the mean of 90-95 % most reactive bases across the library are 1. Then, the top 5-95 % of $r_{\text{icSHAPE\_condition}}$ is scaled to the range of [0, 1] for each transcript independently by 90 % Winsorization (the bottom and top 5 % bases are set to 0 and 1, respectively). This 90 % Winsorization procedure is performed for all positions without 32 bases at the 5′ and 3′ ends of the transcript, due to the difficulties in appropriate mapping. When $c_{\text{icSHAPE\_background, DMSO}}(i,t)$, which does not include cDNA reads that stop at the ith nucleotide, icSHAPE score(i,t) is excluded from the dataset. In addition, due to the bias of read mapping, icSHAPE scores at the very 5′- and 3′-ends are also excluded.

2.3 Log dor ratio computation for BUMHMM

BUMHMM is developed for the robust reactivity computation considering replicate information [17]. BUMHMM employs the log ratio between the drop-off rates at the same nucleotide in a pair of control replicates (log dor ratio, LDR) as an input. Note that this ratio is computed for each position by dividing the raw read count by the read coverage at the same position while the background density appeared in the icSHAPE score computation is the read coverage at one base downstream. Additionally, a sequence information was given since BUMHMM performed construction of null distribution depending on the sequence context.

2.4 Multiple high-throughput structural methodology comparison and computational prediction

To investigate the consistency between multiple HTS, we used the Structure Surfer database [5], in which the structure scores obtained by icSHAPE [15], DMS-Seq [3], PARS [13], and ds/ssRNA-Seq [5] are mapped to human and mouse genomes and combined with ucsc gene ids. From the entire of dataset, we selected parts of studies analyzing human transcriptome, which consisted of two replicates of PARS (rep1 and rep2), DMS-Seq, and the ds/ssRNA-seq dataset. To compare experimental and computational structure analyses, we used ParasoR [14] for prediction of stem probability, which is applicable to long RNAs. Using ParasoR, p_{stem} was computed for each transcript of the hg19 knownGeneMrna dataset downloaded from the UCSC database [18], with a maximal span of $W = 200$. Then, we analyzed the pairwise correlation of the structure scores of experimental and computational analyses, only for transcripts that have the same length in both datasets. The transcripts in which less than 100 nucleotides or less than half of the regions are annotated by structure scores were excluded from the comparison.

After removing the no-score regions, the remaining transcripts were shown in Table 1. Because DMS-Seq and ds/ssRNA-Seq detect the structure score of each nucleotide while other scores correspond to double-strandedness, negative DMS-Seq and ds/ssRNA-Seq scores were applied for the calculation of correlation coefficients. To scale each score into the range of (0, 1), we computed the normalization factors of the minimum and maximum structure scores $smin$ and $smax$, then normalized structure scores as follows.

$$\text{Scaled score} := \frac{\text{Original score} - smin}{smax - smin}$$

2.5 Mapping and quantification of PARS and icSHAPE datasets

For whole transcriptome analyses, we downloaded the PARS score dataset for human transcriptome, as measured in the previous study (GEO accession number is GSE50676) [13]. Normalized read counts of GM12878 native deproteinized replicates with nuclease S1 and V1 treatment (hereafter referred to as S1 and V1, respectively) for two replicates. To remove the influence of indiscriminative multi-mapping reads, we realigned sequencing reads of downloaded fasta files for the set of rRNA repeat unit sequences and human transcriptome. The human transcriptome reference consisted of UCSC Refseq sequences (as of October 7, 2016) and GENCODE transcript sequences (v12), with the reference database constructed as described by Wan et al. [13]. To construct the reference set of the rRNA sequences, a human ribosomal repeating unit (NT_167214.1) was extracted from the NCBI database, and a 5S rRNA sequence (ENST00000364451) was additionally downloaded from the Ensembl database. The process of read counting from the sequencing read data already aligned to the reference sequence was performed by using the Docker image, published with reactIDR. Read counts were then normalized by total counts mapped to the rRNA repeat unit and transcriptome.

Method	ParasoR	PARS rep1	PARS rep2	DMS-Seq	ds/ssRNA-Seq
ParasoR	-	4,327	3,846	48,910	69,248
PARS rep1	-	-	3,716	4,113	4,292
PARS rep2	-	-	-	3,658	3,818
DMS-Seq	-	-	-	-	47,913

Table 1. The number of common transcripts which contain the structure scores observed in both methods.

As a representative of RT stop-based reactivity detection, the icSHAPE dataset was compared with the reference structure and PARS dataset. In this study, the dataset of the previous icSHAPE study for the HEK293T cell was analyzed for the comparison with the *in vitro* PARS dataset (GEO accession number is GSE74353) [19]. We downloaded fastq files generated from three conditions with two replicates, which were DMSO-, *in vitro* NAI-N_3-, and *in vivo* NAI-N_3-treated cells. Then, specific barcodes that were 13-nt in length were removed from 5′-ends of RNA fragments after excluding PCR duplications from the sequence library. RT stop counts were measured for the counts of mapped reads using bowtie2 [20] to the human transcriptome reference constructed for PARS analyses. According to the previous study, it is suggested that a large fraction (more than 20 %) of cDNAs tend to have mismatches at the extreme 3′ end due to the terminal transferase activity of the reverse transcriptase [21]. To confirm the abundance of flanking nucleotides observed in icSHAPE and PARS reads, the local alignment of bowtie2 was also performed. The amount of flanking nucleotides of each length was determined by the number of soft-clipped bases in bam files for each 5′ end.

2.6 Command lines for read mapping

For the PARS dataset, only the first R1 reads truncated to the first 50 bases were mapped with the allowance of one mismatch using bowtie2. A box below shows an example of command lines for the PARS data processing.

```
cat temp.fq \\
| fastx_clipper -a gatcggaagagcggttcagcaggaatgccgag -q33 -m 9 -o
    ↪ temp_trimmed.fq \\
fastx_trimmer -Q33 -l 50 -i temp_trimmed.fq -o temp_trimmed_50.fq
bowtie2 -p 8 -N 1 -x (rRNA index) -U temp_trimmed_50.fq | samtools view -
    ↪ Shb -F 4 - > rRNA.bam
bowtie2 -p 8 -N 1 --local -x (rRNA index) -U temp_trimmed_50.fq | samtools
    ↪ view -Shb -F 4 - > rRNA_local.bam
bowtie2 -p 8 -N 1 -x (transcriptome index) -U temp_trimmed\_50.fq.gz |
    ↪ samtools view -Sb > transcriptome.bam
```

For the icSHAPE dataset, the sequencing reads that are completely duplicated were removed as PCR duplications before barcode truncation, because the number of barcodes incorporated in icSHAPE is large enough that the change to two random fragments to be concatenated with the same barcode is very low ($\frac{1}{4^9}$) [16]. Sequenced reads whose first 13 nucleotides were truncated, were mapped to the reference of transcriptome and rRNA repeat unit using local alignment of bowtie2. The number of reads assigned to each position was counted allowing a single flaking nucleotide at their 5′-end. A box below shows an example of command lines used for icSHAPE data processing.

```
python read_collapse.py --shell --remove --stdout temp.fq > temp\
    ↪ _multiplexed.fq
java -jar trimmomatic-0.36.jar SE -threads 2 -phred33 temp\_multiplexed.fq
    ↪ temp\_read\_trimmed.fq ILLUMINACLIP:../adapters/TruSeq2-SE.fa:2:30:10
    ↪  TRAILING:20 HEADCROP:13
bowtie2 --local -p 8 -x (transcriptome index) -U temp\_read\_trimmed.fq |
    ↪ samtools view -Shb -F 4 - > transcriptome.bam
```

Here is the list of the software tools and their versions used in this study.

1. fastx_toolkit v0.0.13 (`http://hannonlab.cshl.edu/fastx_toolkit/`)
2. bowtie2 v2.2.9 [20]

3. trimmamotic v0.36 [22]

4. samtools v1.3.1 [23]

5. bedtools v2.18 [24]

2.7 Accuracy evaluation of reactIDR structure prediction

As a rRNA structure reference, cryo-EM-based ribosomal structure (PDB ID: 4v6x) was aligned to our reference sequence [25] . To obtain base-to-base correspondence between the reference sequences and the structure, the 5S, 5.8S, 18S, and 28S rRNA reference sequences were aligned to each sequence within the structure dataset, and all bases successfully aligned to the reference were used for the evaluation of classification. Of the four rRNAs in the cryo-EM ribosomal structure, 18S rRNA reference structure was used as the training set for the supervised learning of reactIDR, with the negative data for the minus strand of 18S rRNA. The annotations of secondary structure and base pairings were obtained for this structure using RNAview [26] with the RNApdbee interface [27]. The accessible surface area (ASA) of the ribosome structure was calculated for each nucleotide using NACCESS with default parameter settings [28].

To evaluate the accuracy of structure classification based on reactivity, we determined a receiver operating characteristic (ROC) curve. The number of true positives, true negatives, false positives, and false negatives are referred as TP, TN, FP, and FN. In the ROC curve, the y-axis corresponds to the true positive rates $((TP)/(TP+FN))$ and the x-axis corresponds to the false positive rate $((FP)/(FP+TN))$. P-values were computed to compare the AUC of reactIDR and other scoring methods by pROC library in R with a bootstrap method repeating 100 times [29]. To measure the accuracy of *in silico* structure prediction with or without the structure scores, positive predictive value (PPV) $(TP)/(TP+FP)$, sensitivity (identical to the true positive rates), and Matthews correlation coefficient (MCC) $\frac{(TP\times TN-FP\times FN)}{\sqrt{(TP+FP)(TP+FN)(TN+FP)(TN+FN)}}$ against all the possible base pairs within the transcript. At that time, pseudo-knot and inter-transcript base pairs were converted to the loop structures in the reference. The accuracy of structure prediction based on multiple HTS datasets was also investigated using support vector machine (SVM) and linear discriminant analysis (LDA) as implemented in the scikit-learn library (depending on LIBSVM [30] and LIBLINEAR [31]).

3 Results

3.1 Characteristics of high-throughput structural analysis data

3.2 Read count distribution

To know an applicability of existing case/control comparison methods to HTS data, we examined the PARS data distributions of means and variances of read counts for each base [13]. As a result, the variances were shown to be larger than their means in general, indicating that the read coverage distribution does not follow Poisson distribution (Figure 6). Moreover, it is shown that HTS data consists of many "unmapped" regions so that there are quite a few positions on the line of $y = 2x$, indicating the case of read counts observed only either of replicates. For example, a coordinate point $(x, y) = (1, 2)$ corresponds to the case whose read counts were 0 in one sample and 8 in another. This indicates that HTS data requires a careful treatment for such severe sparsity. Although there is some previous study of zero-inflated negative binomial

modeling for that purpose, IDR is expected to suitable for reactivity estimation by evaluating how read count is enriched in reproducible way or not. Such “no enrichment” is also supposed to be a clue of conformational status in HTS data.

3.3 Multi-comparison between high-throughput structural analyses and *in silico* structure analyses

In this section, we demonstrate the comparison of structure scores of human transcriptome as produced by many different protocols, using the Structure Surfer database [5]. Structure Surfer is a database of transcriptome-wide HTS for multiple species, and we investigated the basic properties of stored HTS dataset.

Figure 1 shows the distribution of structure scores for six HTS. The distributions of structure scores apparently differed between cleavage-based (PARS and ds/ssRNA-Seq) and modification-based approaches (DMS-Seq and icSHAPE). In particular, the score distributions of modification-based approach shows a long tail in one direction. On the other hand, those of PARS and ds/ssRNA-Seq have a median at the middle of distributions because such methods are able to detect discriminative stem and loop regions through the log ratio of the enrichment about single- and double-stranded regions. These differences should be aligned carefully for the cross-comparison between different methodologies, or they might be the cause of spurious inconsistency.

Next, we evaluated the consistency of genome-wide structure analyses on human transcriptome data. We calculated Spearman’s correlation coefficients for the pair of vectors of reactivities assigned to the same location across each human transcript without defect regions. In addition to reactivities measured by experiments, we compared stem probability estimated by ParasoR software *in silico* to confirm the consistency of the thermodynamic folding model and experimental structure analyses. As a result, most correlation coefficients were distributed around 0, indicating random correlation between different studies (Figure 2). PARS replicates, however, showed clearly higher correlation among them compared to others. Also a moderate positive correlation was observed between PARS and ds/ssRNA-Seq. This tendency was robust to the way to select target regions, such as selecting only transcripts depending on their lengths or a specific type of nucleotides (data not shown). In addition, there are a variety on the number of detected transcripts among each HTS, resulting large nonoverlapping for comparison (detailed in the section “Data Processing”).

It should be noted that each HTS data was obtained from different types of cell in the different condition and must show cell-to-cell variation. Hence, this result at least indicated that just a direct comparison of structure scores over multiple HTS exhibited only a weak correlation, despite these studies sharing a common purpose of revealing the structural landscape of human transcriptome. This is a matter of concern because it might be meaningless to analyze the whole landscape of RNA secondary structure over transcriptome if the most of transcripts are truly inconsistent. However, it is not clear whether such incongruence arises from the heterogeneity of human transcriptome as related to the cellular condition or the systematic biases of each method. In fact, RNA secondary structure is known to be variable depending on environmental elements, such as temperature, pH, or interference of binding factors. However, since replicates of HTS showed substantially high correlation in this study, it is considered that most transcripts form a consensus secondary structure that is stable enough to be detected by probing or cleavage.

Moreover, we examined the mismatch occurrence at 5′-end of sequencing read. To evaluate the ratio of flanking base at 5′-end of RNA (or 3′-end of reverse transcribed DNA) fragment, we performed sequence alignment of reads from the PARS and icSHAPE datasets to a reference

of rRNAs. Using bowtie2 with local alignment mode, the number of soft-clipped bases was counted as the number of flanking bases in local alignment mode. As a result, a greater number of reads contained soft-clipped bases in the icSHAPE datasets than the PARS dataset, particularly for the icSHAPE dataset treated with DMSO (Figure 7). This result is consistent with the previous report on flanking nucleotides by the terminal transferase activity [21]. Because it might also reduce the resolution and precision of structure detection, we selected only reads having one or no soft-clip base at 5′-end from icSHAPE datasets in the following analyses.

3.4 Consistency of computational and experimental structure analyses based on IDR classification

Next, the consistency between the PARS dataset and computational structure prediction was examined depending on the IDR-based classification. We tested IDR-based classification that a nucleotide is classified into "S1 (accessible)" and "V1 (stem)" when estimated IDR was lower than 0.01 for S1 and V1 dataset, respectively. We also defined "Neutral" class as the samples included in neither accessible nor stem, or those belonging to both accessible and stem. Figure 8 shows the distribution of stem probabilities for each class based on IDR computed for the S1 and V1 datasets. Consequently, the median of stem probability clearly increased in the order of accessible, neutral, and stem. Although there are still a certain number of inconsistent nucleotides between stem probability and PARS score, they might be influenced by complex secondary structures, such as long-range base pairs or RNA binding protein interaction, as well as the failure of computational prediction.

4 Tables

Comparison of the elapsed time and memory requirement for four rRNAs		
Method	RSS (MB)	Elapsed time (sec)
BUMHMM	360.2	25.8
PROBer (prepare)	10.4	0.24
PROBer (estimate)	715.6	322.0
IDR	100.3	10545
reactIDR (training)	260.3	1955
reactIDR (test)	89.8	15.3
reactIDR (fit)	400.3	2981

Table 2. IDR and reactIDR computes the structure scores for the regular transcripts and their opposite strands using the HTS reads assigned to those regions while BUMHMM and PROBer only considers the reads assigned to the regular transcripts. The *training* process of reactIDR computed the structure scores of only 18S rRNA with its reference structure. The *prepare* process of PROBer constructs the sequence database without HTS data. The *estimate* process of PROBer includes a process of counting the read numbers. IDR indicates the re-implemented IDR computation in reactIDR. reactIDR was set to use 8 processes for acceleration of EM iteration. The experiment was done on our machine (MacBook Pro Late 2013 model with 2.8GHz Intel Core i7 and OS X El Capitan) using Python v3.5.

AUROC for stem and loop classification of the test set of rRNAs based on structure scores						
Method	*in vivo*	*in vitro*	PARS V1	PARS S1	V1 only	S1 only
Count	0.555	0.514	0.702	0.349		
Score	0.600	0.564	† 0.574	† 0.574		
BUMHMM	0.592	0.526	† 0.634	† 0.634		
PROBer	0.645	0.607	† 0.638	† 0.639		
IDR	0.563	0.518	0.664	0.375		
reactIDR	**0.665**	**0.626**	**0.715**	0.420	**0.668**	0.279
Supervised	0.655	0.605	0.609	0.475	0.666	0.278
Unsupervised	0.616	0.517	0.470	0.476	0.667	**0.310**
PARS-based reactIDR	0.617	0.625	-	-	-	-

AUROC for stem and loop classification of the training set of rRNAs based on structure scores						
Method	*in vivo*	*in vitro*	PARS V1	V1 only	PARS S1	S1 only
Count	0.629	**0.578**	0.537		0.544	
Score	0.636	0.578	† 0.556		† 0.556	
BUMHMM	0.574	0.557	† 0.546		† 0.540	
PROBer	0.607	0.550	† 0.559		† 0.549	
IDR	0.607	0.557	0.524		0.534	
reactIDR	0.625	0.535	0.528	0.469	0.519	0.468
Supervised	0.648	0.567	0.550	0.475	0.550	0.468
Unsupervised	0.609	0.535	0.517	**0.486**	0.517	**0.478**
PARS-based reactIDR	0.625	0.521	-	-	-	-

Table 3. The area under the ROC curves (AUROCs) of rRNA structure prediction for the test set by structure scores based on eight kinds of reactivity scoring methods: *Count*, icSHAPE or PARS score (*Score*), BUMHMM [17], PROBer [32], IDR, and reactIDR with the parameters initialized by supervised learning and fitted to each transcript (reactIDR). In addition, the prediction accuracies are shown here for several variants of reactIDR: reactIDR with the parameters initialized by supervised learning and without any further fitting (*Supervised*), that with the parameters fitted to each transcript (*Unsupervised*), and that with the parameters initialized by supervised learning for the PARS dataset then fitted to each transcript. AUROCs for the structure classification of the test set (28S, 5S, and 5-8S rRNAs, shown in the top panel) and training set (18S rRNA, shown in the bottom panel) were computed while the structure status to be enriched (*i.e.*, loop or stem) was set to positive. Each column corresponds to the AUROCs for the prediction of accessible loop sites for *in vivo* and *in vitro* icSHAPE datasets and stem and loop regions for the PARS V1 and S1 dataset, respectively. In addition, we applied the prediction of structure scores without control for the PARS S1 and PARS V1 dataset, shown in *S1 only* and *V1 only*. The AUROCs shown of BUMHMM and PROBer in gray were obtained by the methods not originally designed for the PARS experiments. Those of reactIDR and *Supervised* for 18S rRNA structure prediction are also shown in gray because they used the information of 18S rRNA structure (the training set) through the optimized parameters by supervised learning. In other words, their test set and training set are same. † represents the structure score used for evaluation is common among the case (loop-enriched) and control (stem-enriched) condition. However, even those common structure scores could produce different AUROCs between stem and loop prediction from the PARS S1 and PARS V1 dataset. This is because all nucleotides were not symmetrically classified by assuming the undeterminable nucleotides (*e.g.*, missing data) were the least likely candidates.

AUROC for stem and loop classification with the 28S rRNA reference based on structure scores				
Method	*in vivo*	*in vitro*	PARS V1	PARS S1
Count	0.636	**0.589**	0.528	0.544
Score	0.603	0.563	† **0.555**	† 0.555
BUMHMM	0.584	0.561	† 0.527	† 0.560
PROBer	0.600	0.547	† 0.561	† 0.554
IDR	0.615	0.571	0.511	0.534
reactIDR	0.623	0.569	0.521	0.540
Supervised	**0.650**	0.563	0.535	**0.559**
Unsupervised	0.599	0.543	0.517	0.528

Table 4. The area under the ROC curves (AUROCs) for rRNA structure classification of the test set were computed in a similar way to those of Table3. The training set and test set used here consist of 28S rRNA and other 3 rRNAs, respectively.

5 Figures

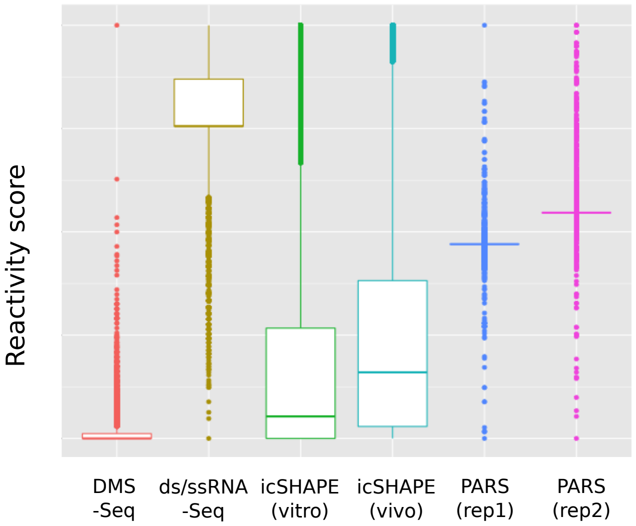

Figure 1. Distribution of structure scores for whole transcriptome of six types of HTS archived in Structure Surfer. Each score distribution is scaled to the range [0, 1].

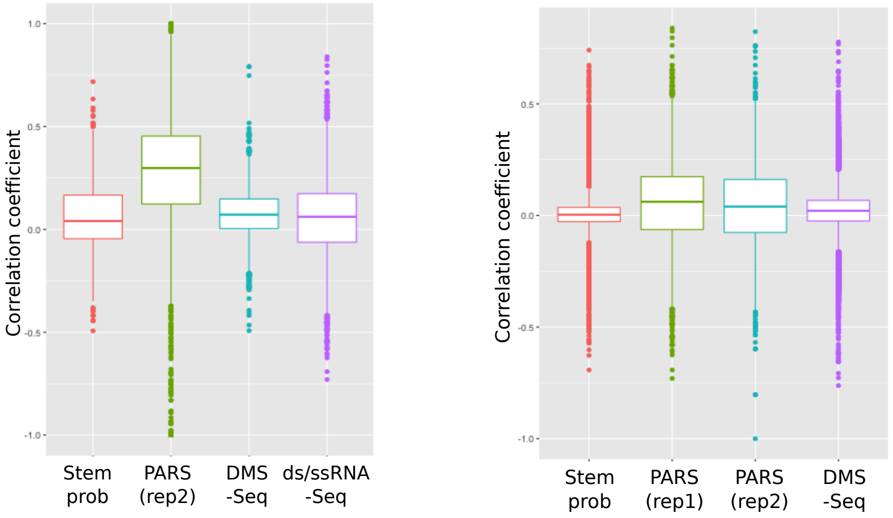

(a) **(b)**

Figure 2. Distribution of correlation coefficients about reactivities between (a) PARS rep1 or (b) ds/ssRNA-Seq and other methods across each human transcript.

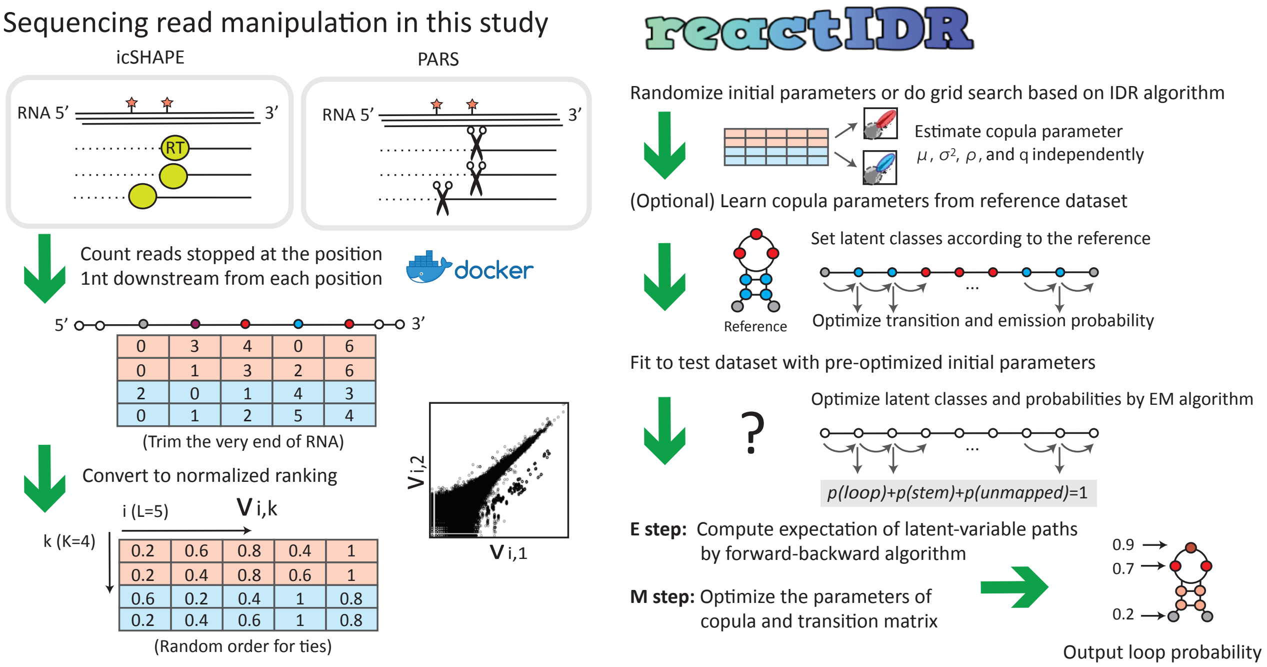

Figure 3. (Left) reactIDR receives the input data consisting of 5′-end read depth repeatedly measured in the case and control conditions. The input data is first converted into the scaled ranking data to adapt the joint cumulative probability distribution of copula model applied in the irreproducible discovery rate (IDR), which discriminates true signals from irreproducible false signals. (Right) In reactIDR, IDR is extendedly combined with the hidden Markov model of the three latent classes (*stem*, *loop*, and *unmapped*) to handle case/control comparison, in which each parameter is optimized to the data and reference structure by expectation-maximization algorithm. Then reactIDR provides the posterior probability of being *loop* and *stem*, which is feasible as a structure score for *in silico* structure prediction.

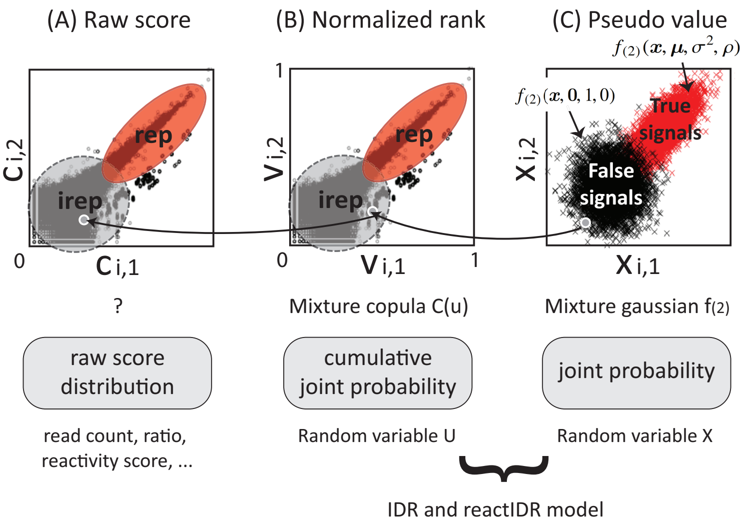

Figure 4. Relationship between raw input value, normalized rank, and pseudo value which corresponds to each normalized rank in IDR.

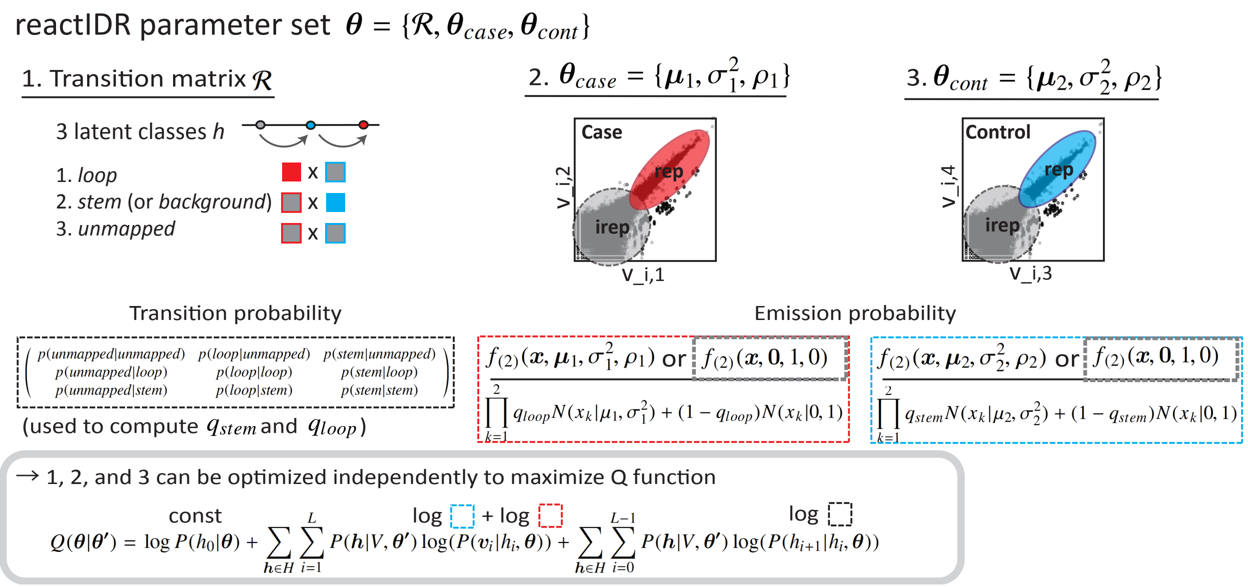

Figure 5. In reactIDR, the parameters to be optimized can be classified into roughly three sets, a transition matrix $\mathcal{R}$ and two copula parameter sets θ_{case} and θ_{cont}. In each M step of EM algorithm, $\mathcal{R}$ is first optimized by the expected transition frequency, then the ratios of reproducible signals q_{loop} and q_{stem} are computed. θ_{case} and θ_{cont} are individually optimized because they are shown in the separated terms in the equation of Q function.

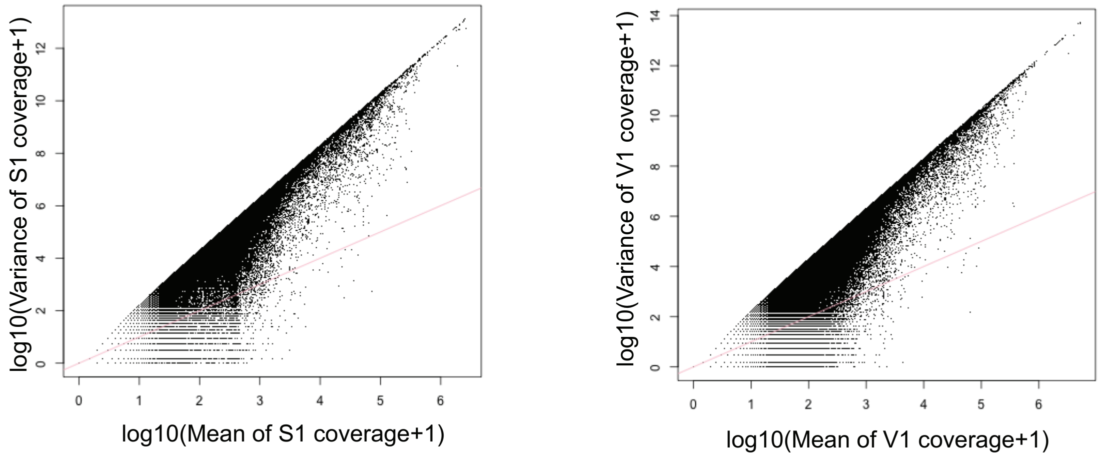

(a) (b)

Figure 6. Relationship between $\log_{10}$ mean and $\log_{10}$ variance of the read count distribution shown in the x- and the y-axis, respectively. (A) and (B) correspond to those of (read count +1) from the PARS S1 and V1 dataset for each nucleotide among the replicates, respectively. Red lines are the $y = x$ lines, which correspond to the equal mean and variance of Poisson distribution.

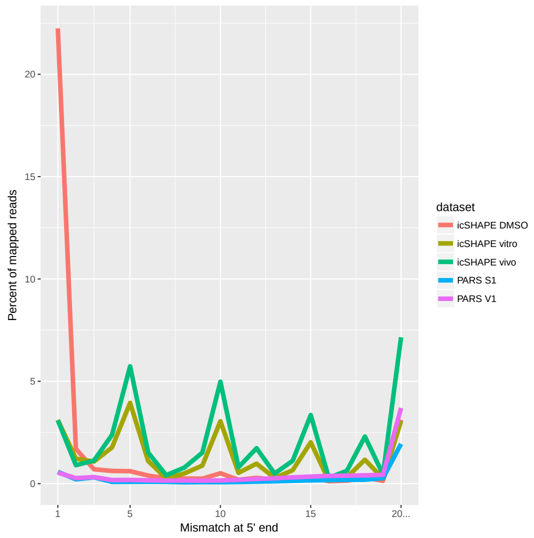

Figure 7. Ratio of sequence reads with at least one flanking bases at their 5′-end. X-axis indicates the number of mismatches (soft-clipped bases) at 5′-end detected by the alignment of bowtie2 with local alignment mode. Y-axis indicates the ratio (percent) of sequence reads whose 5′-ends contain each number of mismatches in icSHAPE treated with DMSO (control), *in vitro* (case), *in vivo* (case), PARS treated with S1 (case), and V1 (control).

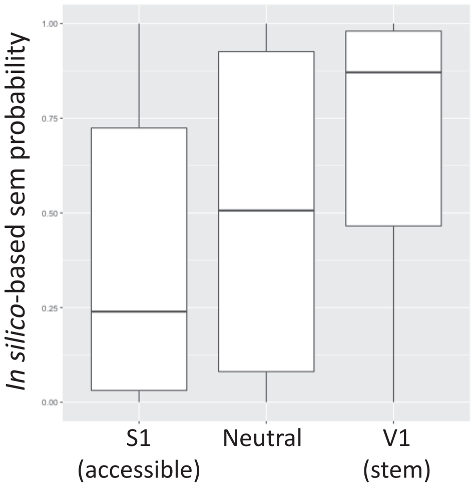

Figure 8. Stem probability distribution computed by *in silico* prediction for the human transcriptome dataset. Three groups shown in x-axis corresponds to the group of being enriched in case, control, or otherwise classified by IDR criteria [1]. Distribution of stem probability based on the IDR-based classification into three classes of accessible, neutral, and structured. Each nucleotide is classified as accessible and structured when estimated IDR is lower than 0.01 for S1 and V1 dataset, respectively. After the classification, the samples included in neither accessible nor structured, or those belonging to both accessible and structured were classified into neutral class.

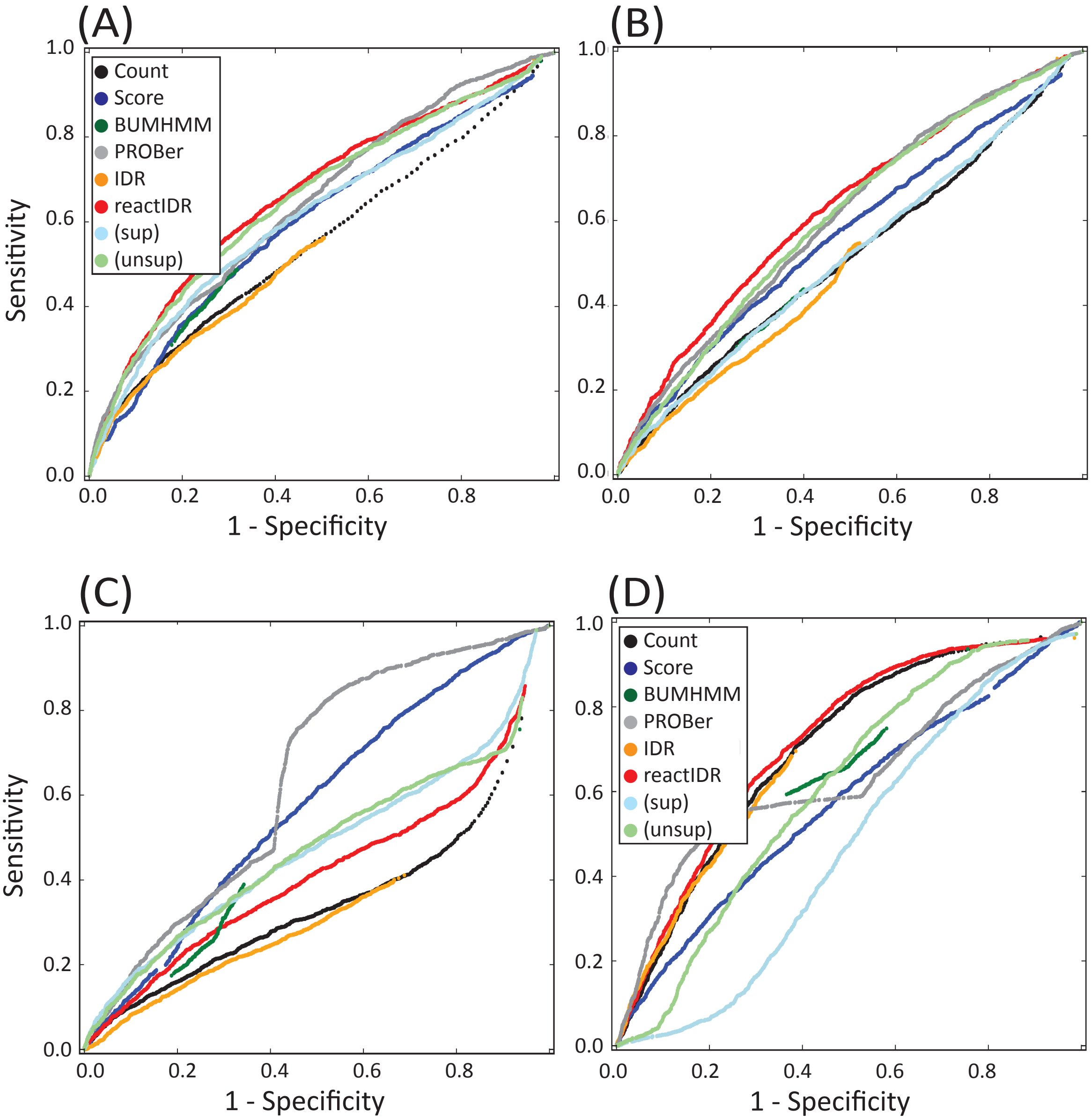

Figure 9. ROC curve of rRNA structure prediction for the test set by structure scores based on eight kinds of reactivity scoring methods: *Count*, icSHAPE or PARS score (*Score*), BUMHMM [17], PROBer [32], IDR, and reactIDR with the parameters initialized by supervised learning and fitted to each transcript (reactIDR), ones obtained by supervised learning (*sup*), and ones fitted to each transcript (*unsup*). Each panel presents the ROC curves for the loop prediction from the *in vivo* icSHAPE dataset (A), *in vitro* icSHAPE dataset (B), the PARS dataset (C), and stem prediction from the PARS dataset (D).

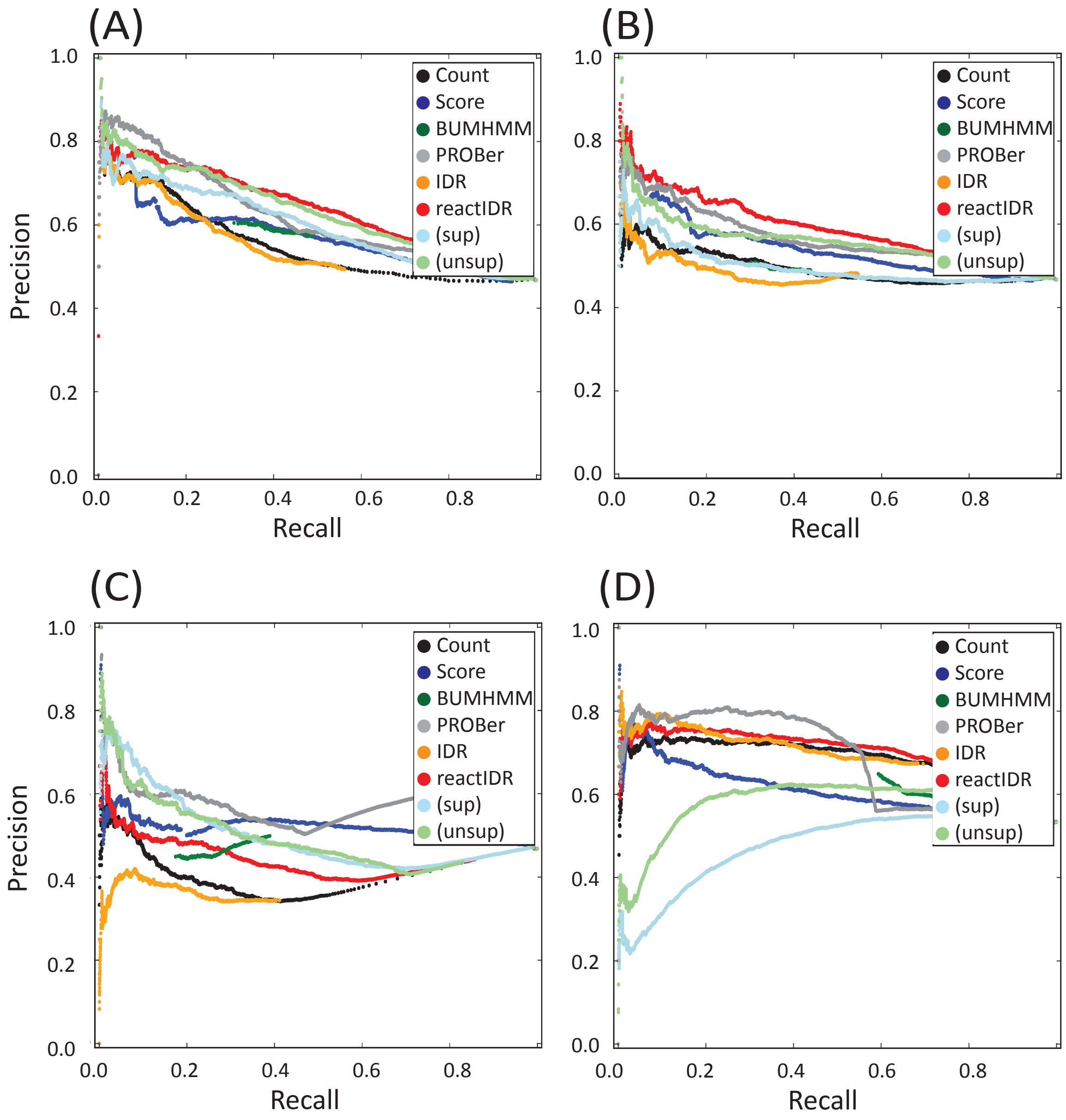

Figure 10. Precision-recall curve of rRNA structure prediction for the test set by structure scores based on eight kinds of reactivity scoring methods as the analysis performed in Figure 9.

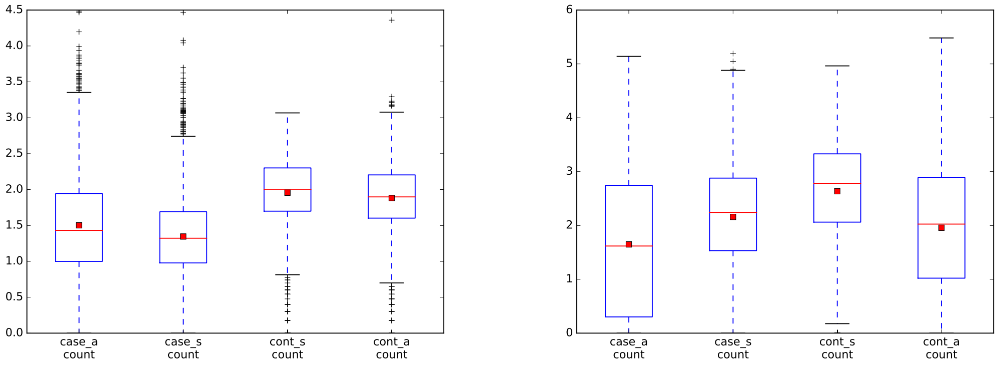

(a) **(b)**

Figure 11. Distribution of $\log_{10}$(Average read depth + 1) at stem and loop regions for case and control samples from (a) the icSHAPE *in vivo* and (b) PARS dataset. case_a and case_s represent a read count distribution in the case samples at loop and stem regions, respectively, while cont_a and cont_s correspond to the those in the control samples.

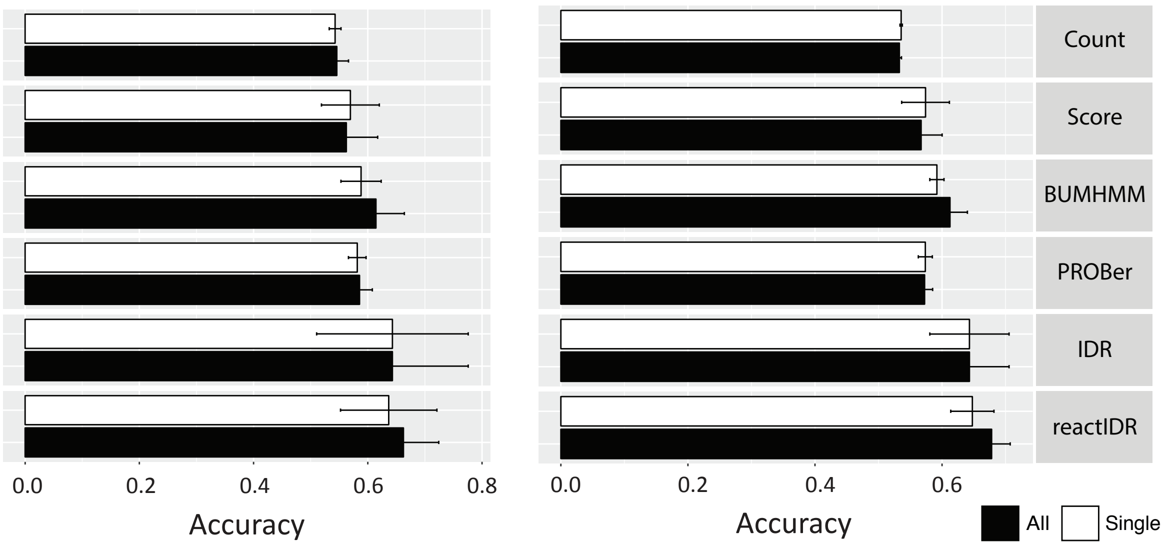

(a) **(b)**

Figure 12. Prediction accuracy of stem/loop structures based on the structure scores computed from individual or combined datasets (the icSHAPE *in vivo* and *in vitro* and PARS *in vitro* datasets) by LDA (a) and SVM (b), with indeterminable scores filtered out. Average and variance of accuracy were computed after 10-fold cross validation.

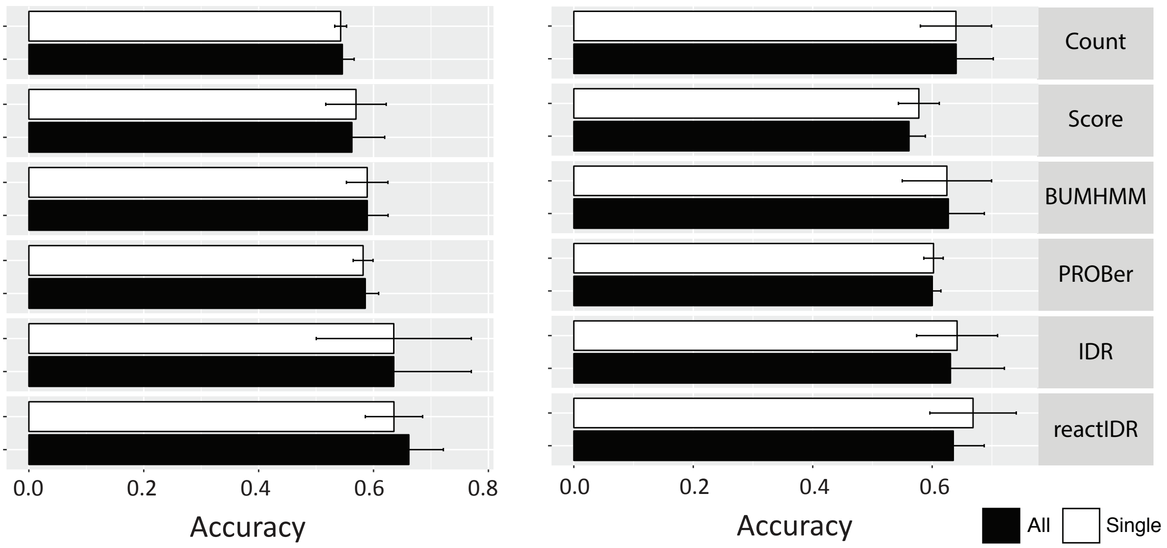

(a) **(b)**

Figure 13. Prediction accuracy of stem/loop structures based on the structure scores computed from individual or combined datasets (the icSHAPE *in vivo* and *in vitro* and PARS *in vitro* datasets) by LDA (a) and SVM (b) without any data filtering. Average and variance of accuracy were computed after 10-fold cross validation just like Figure 12.

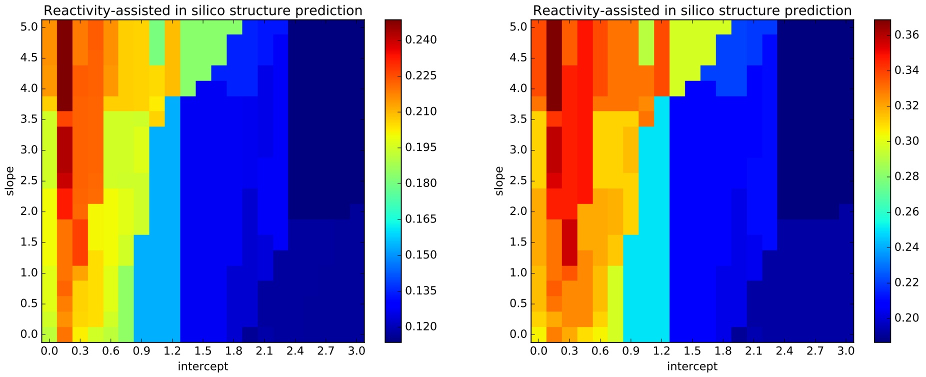

(a) **(b)**

Figure 14. Positive prediction value (PPV) (a) and sensitivity (b) of *in silico* minimum free energy structure prediction of RNAfold for the test set of rRNAs with the guide of PROBer structure scores. The x-axes and y-axes indicate the parameter of slope and intercept for the equation used in RNAfold to compute a pseudo energy, respectively.

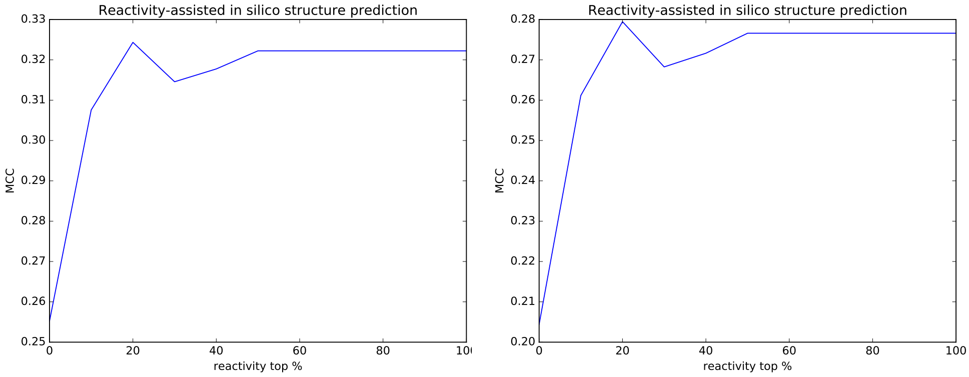

(a) **(b)**

Figure 15. Mathews correlation coefficient (MCC) of *in silico* structure prediction for the test set of rRNAs (a) and 18S rRNAs (b) with the guide of top-ranked structure scores (0 % or no reactivity information, 10 %, 20 %, up to 100 % or all reactivity information).

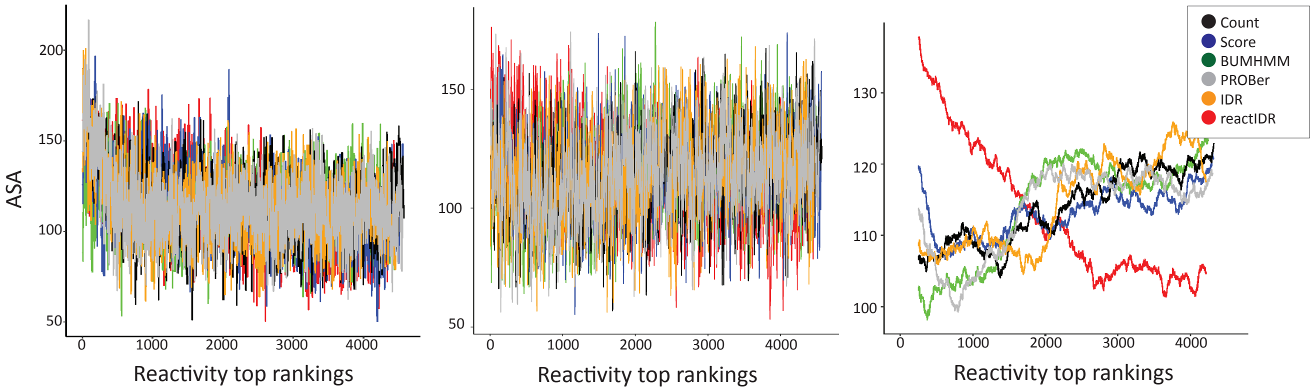

(a) **(b)** **(c)**

Figure 16. Relationship between ASA and structure scores from the icSHAPE *in vivo* (a) and *in vitro* (b and c) icSHAPE dataset. The y-axis corresponds to the averaged ASA within each 10-nt (a and b) and 500-nt (c) sliding window. The x-axes show the ranking order of each nucleotide based on the structure scores in the descending order, in which ties were randomly shuffled.

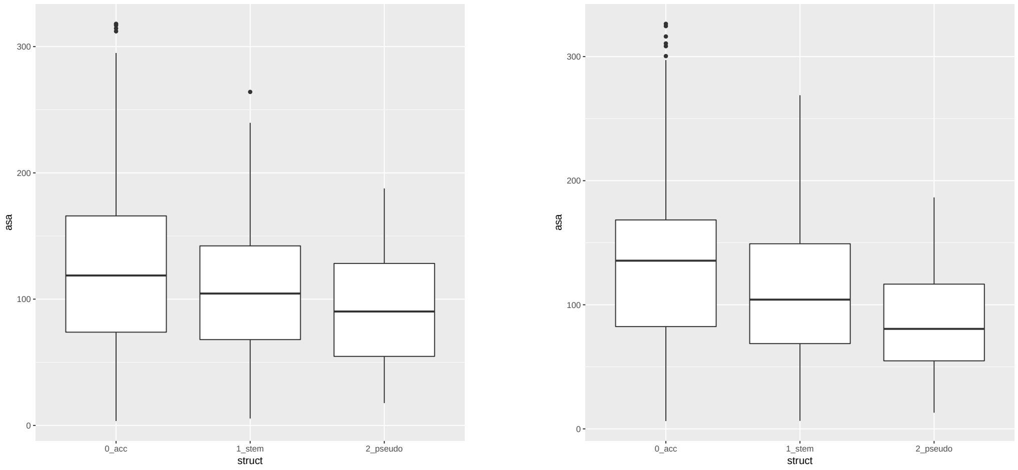

(a) **(b)**

Figure 17. Distribution of ASA of each nucleotide group classified depending on the structure status of secondary structure on the spot, specifically loop, stem, or pseudoknot structures. Each figure indicate ASA distributions of 18S rRNA (a) and other rRNAs (b) obtained from the *in vivo* icSHAPE dataset.

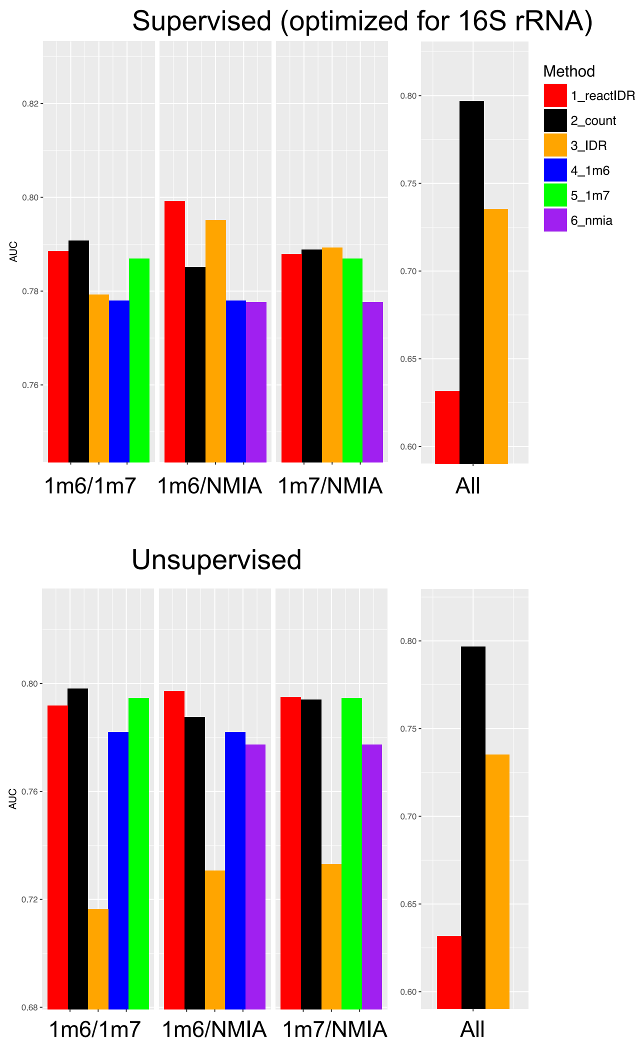

Figure 18. AUROC of *E. coli* rRNA structure prediction for each reactivitiy score based on *E. coli* SHAPE-mutational profiling (MaP) data obtained in Ref. [33] and structure references in CRW database [34]. They published three types of SHAPE-MaP data, in which the reagent 1-methyl-6-nitroisatoic anhydride (1m6), 1-methyl-7-nitroisatoic anhydride (1m7), and N-methylisatoic anhydride (NMIA) was individually applied to rRNAs. Using those data, the prediction of loop regions was performed for 5S and 23S rRNA using the parameters optimized for 16S rRNA in a supervised way, and all rRNAs in an unsupervised way. The performance was evaluated for six types of reactivity scoring methods by calculating AUROCs. To compute the structure scores, reactIDR and IDR took each pair of SHAPE-MaP data as the replicates (shown in the left panels) or all of data as the triplicates (shown in the most right panels). "1m6", "1m7", and "nmia" corresponds to the prediction accuracy using the reactivity scores of the samples treated with 1m6, 1m7, and NMIA, respectively, while "count" corresponds to that using the average of the reactivity scores.

6 Abbreviation

- IDR: irreproducible discovery rate
- HMM: hidden Markov model
- HTS: high-throughput structural analysis
- RT: reverse transcription
- EM: expectation-maximization
- LDR: log dor ratio
- ROC: receiver operating characteristic
- AUROC: area under the receiver operating characteristic curve
- SVM: support vector machine
- LDA: linear discriminant analysis
- PARS: parallel analysis of RNA structure
- SHAPE: selective 2′-hydroxyl acylation analyzed by primer extension
- DMS: dimethyl sulfate
- MaP: mutational profiling
- 1m6: 1-methyl-6-nitroisatoic anhydride
- 1m7: 1-methyl-7-nitroisatoic anhydride
- NMIA: N-methylisatoic anhydride

References

[1] Qunhua Li, James B Brown, Haiyan Huang, and Peter J Bickel. Measuring reproducibility of high-throughput experiments. *The annals of applied statistics*, 5(3):1752–1779, 2011.

[2] Julius B Lucks, Stefanie A Mortimer, Cole Trapnell, Shujun Luo, Sharon Aviran, Gary P Schroth, Lior Pachter, Jennifer A Doudna, and Adam P Arkin. Multiplexed RNA structure characterization with selective 2′-hydroxyl acylation analyzed by primer extension sequencing (SHAPE-Seq). *Proceedings of the National Academy of Sciences*, 108(27): 11063–11068, 2011.

[3] Silvi Rouskin, Meghan Zubradt, Stefan Washietl, Manolis Kellis, and Jonathan S Weissman. Genome-wide probing of RNA structure reveals active unfolding of mRNA structures in vivo. *Nature*, 505(7485):701–705, 2014.

[4] Michael Kertesz, Yue Wan, Elad Mazor, John L Rinn, Robert C Nutter, Howard Y Chang, and Eran Segal. Genome-wide measurement of RNA secondary structure in yeast. *Nature*, 467(7311):103–107, 2010.

[5] Nathan D Berkowitz, Ian M Silverman, Daniel M Childress, Hilal Kazan, Li-San Wang, and Brian D Gregory. A comprehensive database of high-throughput sequencing-based RNA secondary structure probing data (Structure Surfer). *BMC bioinformatics*, 17(1):1, 2016.

[6] William A Ziehler and David R Engelke. Probing RNA structure with chemical reagents and enzymes. *Current protocols in nucleic acid chemistry*, pages 6–1, 2001.

[7] Fan Li, Qi Zheng, Lee E Vandivier, Matthew R Willmann, Ying Chen, and Brian D Gregory. Regulatory impact of RNA secondary structure across the Arabidopsis transcriptome. *The Plant Cell*, 24(11):4346–4359, 2012.

[8] Hiroko Ohmiya, Morana Vitezic, Martin C Frith, Masayoshi Itoh, Piero Carninci, Alistair RR Forrest, Yoshihide Hayashizaki, and Timo Lassmann. RECLU: a pipeline to discover reproducible transcriptional start sites and their alternative regulation using capped analysis of gene expression (CAGE). *BMC genomics*, 15(1):1, 2014.

[9] Geoffrey P Harrison, Matthew S Mayo, Eric Hunter, and Andrew ML Lever. Pausing of reverse transcriptase on retroviral RNA templates is influenced by secondary structures both 5′ and 3′ of the catalytic site. *Nucleic acids research*, 26(14):3433–3442, 1998.

[10] Radford M Neal and Geoffrey E Hinton. A view of the EM algorithm that justifies incremental, sparse, and other variants. In *Learning in graphical models*, pages 355–368. Springer, 1998.

[11] Gábor Csárdi, Alexander Franks, David S Choi, Edoardo M Airoldi, and D Allan Drummond. Accounting for experimental noise reveals that mRNA levels, amplified by post-transcriptional processes, largely determine steady-state protein levels in yeast. *PLoS genetics*, 11(5):e1005206, 2015.

[12] Michael Kertesz, Nicola Iovino, Ulrich Unnerstall, Ulrike Gaul, and Eran Segal. The role of site accessibility in microRNA target recognition. *Nature genetics*, 39(10):1278–1284, 2007.

[13] Yue Wan, Kun Qu, Qiangfeng Cliff Zhang, Ryan A Flynn, Ohad Manor, Zhengqing Ouyang, Jiajing Zhang, Robert C Spitale, Michael P Snyder, Eran Segal, et al. Landscape and variation of RNA secondary structure across the human transcriptome. *Nature*, 505 (7485):706–709, 2014.

[14] Risa Kawaguchi and Hisanori Kiryu. Parallel computation of genome-scale RNA secondary structure to detect structural constraints on human genome. *BMC bioinformatics*, 17(1):1, 2016.

[15] Robert C Spitale, Ryan A Flynn, Qiangfeng Cliff Zhang, Pete Crisalli, Byron Lee, Jong-Wha Jung, Hannes Y Kuchelmeister, Pedro J Batista, Eduardo A Torre, Eric T Kool, et al. Structural imprints in vivo decode RNA regulatory mechanisms. *Nature*, 519(7544): 486–490, 2015.

[16] Ryan A Flynn, Qiangfeng Cliff Zhang, Robert C Spitale, Byron Lee, Maxwell R Mumbach, and Howard Y Chang. Transcriptome-wide interrogation of RNA secondary structure in living cells with icSHAPE. *Nature protocols*, 11(2):273–290, 2016.

[17] Alina Selega, Christel Sirocchi, Ira Iosub, Sander Granneman, and Guido Sanguinetti. Robust statistical modeling improves sensitivity of high-throughput RNA structure probing experiments. *Nature Methods*, 14(1), 2017.

[18] Kate R Rosenbloom, Joel Armstrong, Galt P Barber, Jonathan Casper, Hiram Clawson, Mark Diekhans, Timothy R Dreszer, Pauline A Fujita, Luvina Guruvadoo, Maximilian Haeussler, et al. The UCSC genome browser database: 2015 update. *Nucleic acids research*, 43(D1):D670–D681, 2015.

[19] Zhipeng Lu, Qiangfeng Cliff Zhang, Byron Lee, Ryan A Flynn, Martin A Smith, James T Robinson, Chen Davidovich, Anne R Gooding, Karen J Goodrich, John S Mattick, et al. RNA Duplex Map in Living Cells Reveals Higher-Order Transcriptome Structure. *Cell*, 165(5):1267–1279, 2016.

[20] Ben Langmead and Steven L Salzberg. Fast gapped-read alignment with Bowtie 2. *Nature methods*, 9(4):357–359, 2012.

[21] Lukasz Jan Kielpinski and Jeppe Vinther. Massive parallel-sequencing-based hydroxyl radical probing of RNA accessibility. *Nucleic acids research*, 42(8):e70, 2014.

[22] Anthony M Bolger, Marc Lohse, and Bjoern Usadel. Trimmomatic: a flexible trimmer for Illumina sequence data. *Bioinformatics*, 30(15):2114–2120, 2014.

[23] Heng Li, Bob Handsaker, Alec Wysoker, Tim Fennell, Jue Ruan, Nils Homer, Gabor Marth, Goncalo Abecasis, and Richard Durbin. The sequence alignment/map format and SAMtools. *Bioinformatics*, 25(16):2078–2079, 2009.

[24] Aaron R Quinlan and Ira M Hall. BEDTools: a flexible suite of utilities for comparing genomic features. *Bioinformatics*, 26(6):841–842, 2010.

[25] Andreas M Anger, Jean-Paul Armache, Otto Berninghausen, Michael Habeck, Marion Subklewe, Daniel N Wilson, and Roland Beckmann. Structures of the human and Drosophila 80S ribosome. *Nature*, 497(7447):80, 2013.

[26] Huanwang Yang, Fabrice Jossinet, Neocles Leontis, Li Chen, John Westbrook, Helen Berman, and Eric Westhof. Tools for the automatic identification and classification of RNA base pairs. *Nucleic acids research*, 31(13):3450–3460, 2003.

[27] Maciej Antczak, Tomasz Zok, Mariusz Popenda, Piotr Lukasiak, Ryszard W Adamiak, Jacek Blazewicz, and Marta Szachniuk. RNApdbee—a webserver to derive secondary structures from pdb files of knotted and unknotted RNAs. *Nucleic acids research*, 42(W1): W368–W372, 2014.

[28] SJ Hubbard and JM Thornton. Naccess: Department of biochemistry and molecular biology, university college london. *Software available at http://www.bioinf.manchester.ac.uk/naccess/nacdownload.html*, 1993.

[29] Xavier Robin, Natacha Turck, Alexandre Hainard, Natalia Tiberti, Frédérique Lisacek, Jean-Charles Sanchez, and Markus Müller. proc: an open-source package for r and s+ to analyze and compare roc curves. *BMC bioinformatics*, 12(1):77, 2011.

[30] Chih-Chung Chang and Chih-Jen Lin. LIBSVM: a library for support vector machines. *ACM transactions on intelligent systems and technology (TIST)*, 2(3):27, 2011.

[31] Rong-En Fan, Kai-Wei Chang, Cho-Jui Hsieh, Xiang-Rui Wang, and Chih-Jen Lin. LIB-

LINEAR: A library for large linear classification. *Journal of machine learning research*, 9:1871–1874, 2008.

[32] Bo Li, Akshay Tambe, Sharon Aviran, and Lior Pachter. PROBer Provides a General Toolkit for Analyzing Sequencing-Based Toeprinting Assays. *Cell Systems*, 4(5):568–574, 2017.

[33] Nathan A Siegfried, Steven Busan, Greggory M Rice, Julie AE Nelson, and Kevin M Weeks. RNA motif discovery by SHAPE and mutational profiling (SHAPE-MaP). *Nature methods*, 11(9):959–965, 2014.

[34] Jamie J Cannone, Sankar Subramanian, Murray N Schnare, James R Collett, Lisa M D'Souza, Yushi Du, Brian Feng, Nan Lin, Lakshmi V Madabusi, Kirsten M Müller, et al. The comparative RNA web (CRW) site: an online database of comparative sequence and structure information for ribosomal, intron, and other RNAs. *BMC bioinformatics*, 3(1): 1, 2002.
